# Barriers and facilitators to utilizing digital health technologies by healthcare professionals

**DOI:** 10.1038/s41746-023-00899-4

**Published:** 2023-09-18

**Authors:** Israel Júnior Borges do Nascimento, Hebatullah Abdulazeem, Lenny Thinagaran Vasanthan, Edson Zangiacomi Martinez, Miriane Lucindo Zucoloto, Lasse Østengaard, Natasha Azzopardi-Muscat, Tomas Zapata, David Novillo-Ortiz

**Affiliations:** 1 Division of Country Health Policies and Systems (CPS), World Health Organization Regional Office for Europe, Copenhagen, 2100 Denmark; 2 Pathology and Laboratory Medicine, Medical College of Wisconsin, Milwaukee, WI 53226-3522 USA; 3 Department of Sport and Health Science, Techanische Universität München, Munich, 80333 Germany; 4 Physical Medicine and Rehabilitation Department, Christian Medical College, Vellore, Tamil Nadu 632004 India; 5 Department of Social Medicine and Biostatistics, Ribeirão Preto Medical School, University of São Paulo, Ribeirão Preto, São Paulo, 14049-900 Brazil; 6 Centre for Evidence-Based Medicine Odense (CEBMO) and Cochrane Denmark, Department of Clinical Research, University Library of Southern Denmark, Odense, 5230 Denmark

**Keywords:** Health occupations, Public health

## Abstract

Digital technologies change the healthcare environment, with several studies suggesting barriers and facilitators to using digital interventions by healthcare professionals (HPs). We consolidated the evidence from existing systematic reviews mentioning barriers and facilitators for the use of digital health technologies by HP. Electronic searches were performed in five databases (Cochrane Database of Systematic Reviews, Embase^®^, Epistemonikos, MEDLINE^®^, and Scopus) from inception to March 2023. We included reviews that reported barriers or facilitators factors to use technology solutions among HP. We performed data abstraction, methodological assessment, and certainty of the evidence appraisal by at least two authors. Overall, we included 108 reviews involving physicians, pharmacists, and nurses were included. High-quality evidence suggested that infrastructure and technical barriers (Relative Frequency Occurrence [RFO] 6.4% [95% CI 2.9–14.1]), psychological and personal issues (RFO 5.3% [95% CI 2.2–12.7]), and concerns of increasing working hours or workload (RFO 3.9% [95% CI 1.5–10.1]) were common concerns reported by HPs. Likewise, high-quality evidence supports that training/educational programs, multisector incentives, and the perception of technology effectiveness facilitate the adoption of digital technologies by HPs (RFO 3.8% [95% CI 1.8–7.9]). Our findings showed that infrastructure and technical issues, psychological barriers, and workload-related concerns are relevant barriers to comprehensively and holistically adopting digital health technologies by HPs. Conversely, deploying training, evaluating HP’s perception of usefulness and willingness to use, and multi-stakeholders incentives are vital enablers to enhance the HP adoption of digital interventions.

## Introduction

Recent developments in health technology have positively affected multiple and essential sectors of the economy, especially the healthcare sector, by providing solutions that guarantee the exchange of medical knowledge and information and establish long-lasting health outcomes^1,2^. Digital health technologies, such as wearables devices, computerized decision support systems, and telemedicine improve the technical performance and satisfaction of healthcare employees, demonstrate potential to decrease direct and indirect costs of medical services, and enhance the quality of delivered care^3^. Worldwide, using digital solutions in practice seems inevitable, with modality-specific prevalence (e.g., 50.8% for telemedicine, 89.9% for electronic health records, and 91.9% for social media platforms)^4–6^. However, the prevalence of use might be even higher, as no previous study has collated and assessed the overall prevalence of using digital health technologies by healthcare providers. Likewise, several studies have suggested that ethnicity, race, geographic location, age, and medical specialty directly interfere in the adoption of technology use, evidencing the importance of understanding variables accounting for the digital divide and disparity of access^7–9^.

Several barriers to healthcare’s overall quality, transparency, and efficiency naturally arise during or following the creation, implementation, and maintenance of digital health technologies. Therefore, during the design of any health-related project, it is essential to identify and quanti-qualitatively analyze its risks and facilitators, enhancing the likelihood of obtaining favorable outcomes and optimizing the chances of success. The efficient implementation of digital technologies, characterized by proper implementation of a systematic management approach, including strategic planning, resource allocation, and control and evaluation processes, is fundamental to refining healthcare services, equipment, and technologies^10–12^. In reaction to these aforementioned elements, multiple efforts have strengthened healthcare systems through employing DHTs for healthcare professionals and stakeholders from low-, middle-, and high-income countries. For instance, the World Health Organization (WHO) endorsed in the 73rd World Health Assembly the institution of the Global Strategy on Digital Health 2020–2025, in which four guiding principles rely on the acknowledgment that the institutionalization of digital health in a national system requires a decision and commitment by countries, recognition that successful digital technologies require an integrated strategy, promotion of the appropriate use of digital interventions for health, and recognition of the urgent need to address the major impediments faced by least-developed countries implementing digital health technologies^13^. Furthermore, the Regional Digital Health Action Plan for the WHO European Region 2023–2030 has a critical regional focus area on strengthening digital literacy skills and capacity-building in the general population, with particular attention to the health workforce, for the use of digital health services and disease prevention and management^14^. Due to these global actions, numerous studies have focused on assessing barriers to and facilitators for many technologies^15–17^.

To date, hundreds of clinical trials based on specific technologies applied to the healthcare professionals’ environments have assessed the implementation of digital interventions in the healthcare system, while several systematic reviews have combined these publications, evidencing their effectiveness, safety, and feasibility. However, a summary of enablers and restraints to healthcare professionals’ coordinated and integrated use of digital health technologies has not been published yet, making the current evidence dispersed, misused, and overlooked. Therefore, in this overview of systematic reviews and semantic-based occurrence meta-analysis, we report all published evidence from existing systematic reviews covering and mentioning barriers and facilitators to the solid use of digital health technologies by healthcare providers.

## Results

### Study selection and characteristics

**Fig. 1:**
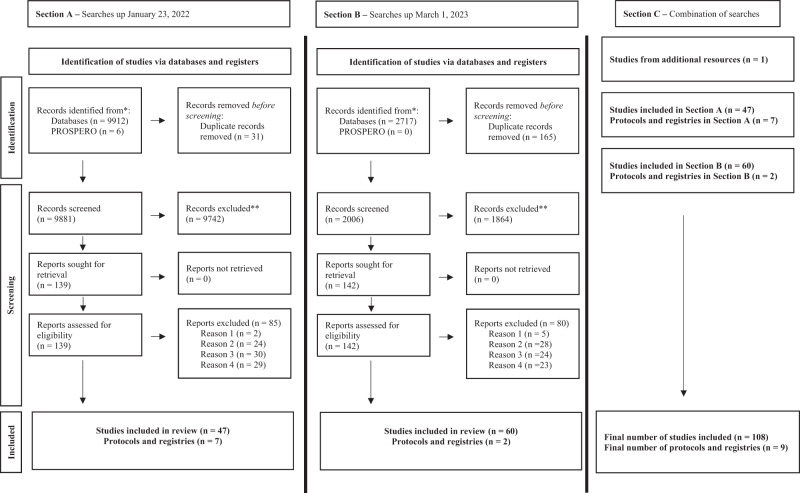
PRISMA flow chart diagram. Reason 1—wrong intervention or platform was unclear. Reason 2—the study did not provide any relevant outcome influencing healthcare providers. Reason 3—targeted population was not healthcare providers. Reason 4—study design used did not match our inclusion criteria.

Our database and PROSPERO search are shown in Fig. 1. Our January 21, 2022 search retrieved 9,912 records, of which 139 underwent full-text review (Fig. 1, section A). Based on the inclusion and exclusion criteria, 47 studies and seven ongoing studies were included. On March 1, 2023, 2,717 new publications were identified through an additional database search (Fig. 1, section B). Of those, 142 studies were shortlisted for full-text assessment, and 60 reviews were added to our umbrella review. Two additional ongoing studies or protocols were identified. In total, this overview of systematic reviews included 108 primary systematic reviews and nine ongoing studies (Fig. 1, section C).^18–125^ One study was identified from alternative resources.^64^ Justification for the exclusion of 165 studies is presented in Supplementary Information 1 (pp 2–7**)**. Included study characteristics are characterized in Table 1 and Table 2. One study is pending classification as it required translation. No additional data needed to be requested from the corresponding authors.

**Table 1:** Main characteristics of included studies evaluating the impact of digital health solutions on health workers (*n* = 108).

Study ID	Publication Year	Journal	Number of Included Databases	Which Databases	Study Objective	Number of Included Studies
Adepoju 2017	2017	JMIR mHealth uHealth	5	PubMed, CINAHL, Web of Science, Cochrane Library of Systematic Reviews, and Grey Literature	To synthesize evidence on the use of mHealth for point-of-care decision support and improved quality of care by health care workers in Africa	22
Addotey-Delove 2023	2023	Int J Env. Res. and Public Health	2	PubMed and Scopus	To identify and examine empirical evidence to answer the research question “what factors have impacted (enabled or impeded) adoption of mHealth by healthcare workers in developing countries?”	85
Alkhaldi 2023	2023	JMIR mHealth and uHealth	4	MEDLINE, Scopus, CINAHL, and PsycINFO	To classify and evaluate interventions aimed at encouraging HCPs to prescribe mHealth apps	11
Al Bawashdeh 2022	2022	Sensors	9	Google Scholar, Science Direct, Emerald, Wiley, PubMed, Springer, MDPI, IEEE, and Scopus	To accumulate existing knowledge about the factors that influence medical professionals to adopt IoT applications in the healthcare sector	22
Agarwal 2015	2015	Trop Med Int Health	5	MEDLINE, Embase, Global Health, Google Scholar, and Scopus	To review and synthesize the evidence on the feasibility and effectiveness of mobile-based services for healthcare delivery by front line health workers	42
Amoakoh-Coleman 2016	2016	J Med Internet Res	5	Cochrane Library of Systematic Reviews, PubMed, Embase, Global Health Library, and PopLine	To evaluate the effectiveness of mHealth interventions targeting health care workers to improve maternal and neonatal outcomes in LMIC	19
Arsad 2023	2023	J of Health Research	5	Ovid, Web Of Science, PubMed, SAGE and EBSCOhost	To identify and review the impact of eHealth applications (apps) on healthcare interventions	10
Aslani 2022	2022	Int Cardiovascular Research Journal	3	PubMed, Scopus, and Web of Science	To identify the advantages and disadvantages of using telecardiology and to provide solutions for its successful implementation based on the obtained results	30
Avoka 2022	2022	Trop Med Int Health	4	PubMed, Embase, Cochrane Register and CINAHL Plus	To review the evidence on interventions to improve obstetric emergency referral decision making, communication and feedback between health facilities in sub-Saharan Africa	14
Balusxkek 2022	2022	BMC Health Services Research	4	MEDLINE, Embase, APA PsycINFO, and CINAHL	To identify and categorize challenges experienced and/or perceived by practitioners	5
Bervell 2019	2019	Soc Sci Med	6	Google Scholar, Springer, Global Health, PubMed, IEEE Xplore, Science Direct	To provide an in-depth look at e-health and m-health utilization in SSA countries, together with the opportunities they offer and the challenges in their trends of usage	61
Boonstra 2010	2010	BMC Heal Serv Res	4	Science, EBSCO, PubMed, and Cochrane Library of Systematic Reviews	To identify, categorize, and analyze barriers perceived by physicians to the adoption of EMRs in order to provide implementers with beneficial intervention options	22
Brommeyer 2023	2023	Int J of Medical Informatics	8	Scopus, ProQuest, Web of Science, ACM Digital Library, CINAHL, PubMed, Google Scholar and ProQuest Dissertations	To present and discuss the findings from a scoping review identifying: 1) competencies required for health service managers leading the implementation and transformation of informatics and digital technology in the health sector; and 2) factors that are critical to building the management workforce capacity in the era of health informatics and digital health	19
Braun 2013	2013	PLoS ONE	7	PubMed/MEDLINE, CAB Global Health, Web of Science, INSPEC, WHO publication database, Health UnBound Content Library, and Royal Tropical Institute Resource Database	To review the evidence for the use of mobile technology by community health workers to identify opportunities and challenges for strengthening health systems in resource-constrained settings	25
Brewster 2014	2014	J Adv Nurs	7	Assia, AMED, British Nursing Index, CINAHL, Embase, MEDLINE, and Web of Knowledge	To synthesize qualitative and quantitative evidence of front-line staff acceptance of the use of telehealth technologies for the management of Chronic Obstructive Pulmonary Disease and Chronic Heart Failure	10
Brown 2020	2020	J Clin Nurs	5	CINAHL, Embase, PsychINFO, MEDLINE, and PubMed	To evaluate and synthesize the evidence regarding the development of digital capability in nurses and the strategies that support effective integration of digital skills into the workplace	17
Calleja 2022	2022	Rural and Remote Health	6	CINAHL, MEDLINE, Nursing & Allied Health (Proquest), PubMed, Joanna Briggs Institute Evidence Based Practice, and Embase	To determine the existence and characteristics of telehealth education in rural and remote setting; evaluate current telehealth education models and resources; establish the quality of education provided through telehealth along with the facilitators or enablers of a successful service; and develop recommendations for supporting and developing an education model for rural and remote health practitioners through telehealth	60
Cansdale 2022	2022	BMJ Open	3	PubMed, CINAHL and Global Health	To evaluate which mHealth tools have been reported to birth outcomes in the delivering room in LMICs and document their reported advantages and drawbacks	21
Cartolovni 2022	2022	Int J Med Inform	6	PubMed, Web of science, Ovid, Scopus, IEEE Xplore, EBSCO Search (Academic Search Premier, CINAHL, PSYCINFO, APA PsycArticles, ERIC)	To surface the underlying ethical and legal but also social implications (ELSI) that have been overlooked in recent reviews while deserving equal attention in the development stage, and certainly ahead of implementation in healthcare	94
Celes 2018	2018	Pan American J of Public Health	3	Virtual Health Library, PubMed, and Google Scholar	To identify telehealth initiatives described in the literature as a strategy for national health policies	21
Cen 2022	2022	BMJ Open	6	PubMed, Scopus, MEDLINE, Web of Science, ScienceDirect, and China National Knowledge Infrastructure	To determine how eHealth was adopted in pharmaceutical care (PC), the outcome reported and the contextual factors	43
Chan 2018	2018	J. Med. Internet Res.	4	MEDLINE, Embase, CINAHL, and InfoSci Journals	To examine the utilization of SNSs for communication among health professionals in frontline clinical practice, professional networks, and education and training to identify areas for future health communication research	33
Chen 2022	2022	Frontiers in Medicine	4	PubMed, Embase, IEEE Xplore, and Web of Science	To investigate clinical AI acceptance among physicians and medical students around the world to provide implementation guidance	60
Christensen 2020	2020	J Psychiatr Ment Health Nurs	8	PubMed, Academic Search Premiere, CINHAL, Scopus, PyscINFO, Web of Science, Sociological Abstracts, and Embase	To conduct a systematic review of the existing research literature, focusing on patients’ and providers’ experiences of VCs used in the treatment of patients 60+ years with unipolar depression	21
da Costa 2020	2020	Telemedicine. e-Health	5	PubMed/MEDLINE, Virtual Health Library, CINAHL, Scopus, and Web of Science	To collect information regarding the inclusion of the application of TD tools in the public dental health services	24
Davis 2014	2014	Telemedicine. e-Health	3	MEDLINE, IEEE Xplore, and Compendex	To explore the acceptability and feasibility of RMT use in routine adult patient care, from the perspectives of primary care clinicians, administrators, and clinic staff	15
de Grood 2016	2016	J Multidisc Healthcare	3	MEDLINE, Embase, and PsycINFO	To summarize the current literature identifying barriers and opportunities that facilitate adoption of e-health technology by physicians.	74
Drissi 2021	2021	Telemedicine. e-Health	5	IEEE Xplore, ACM, ScienceDirect, Scopus, and PubMed	To identify available e-mental health interventions, reported in the literature, that are developed for HCWs during the COVID-19 pandemic	11
Dutta 2020	2020	Medicine	5	PubMed, Web of Science, Scopus, Cochrane Library of Systematic Reviews, and ProQuest	To explore and identify the potential barriers perceived by physicians in the adoption of EMR	26
Early 2019	2019	Health Promot Pract	7	Web of Science, CINAHL, PubMed, MEDLINE, Academic Search Complete, Cochrane Library of Systematic Reviews, and Google Scholar	To identify and describe over ten years of studies on the use, effectiveness, and potential of mHealth involving Community Health Workers	64
Ebneter 2022	2022	Swiss Med Wkly	4	PubMed, MEDLINE, Cochrane Library of Systematic Reviews, and Scopus	To analyze the needs, elements of feasibility, and reasons for acceptance or possible barriers before the implementation of a telemedicine intervention in Switzerland	31
Emmett 2022	2022	Journal of Clin Nursing	7	TRIP, CINAHL, EMCARE, MEDLINE, Scopus, PsychINFO, and EMBASE	To identify and explore the experiences of health professionals towards using mobile electrocardiogram (ECG) technology	6
Ferdousi 2021	2021	Int Nurs Rev	7	MEDLINE, Embase, Cochrane Library of Systematic Reviews, CINAHL, Scopus, Web of Science and Farsi Databases	To evaluate the attitudes of Iranian nurses towards clinical information systems in nursing practice	17
Fletcher 2023	2023	BMC Primary Care	3	MEDLINE, HMIC, and Web of Science	To identify the available evidence on the use of eCDS tools by health professionals in general practice in relation to their impact on workload and workflow	95
Ftouni 2022	2022	BMC Med Inform Decis Mak	7	PubMed, Scopus, Web of Science, Academic Search Complete, CINAHL, Embase, and Science Direct	To explore the barriers and challenges of telemedicine use during the pandemic and to propose solutions for improving future use	
Gagnon 2012	2012	J Med Syst	14	MEDLINE, Embase, CINAHL, Cochrane Library of Systematic Reviews, DARE, Biosis Previews, PsycINFO, Current Content, HSTAT, Dissertation Abstracts, ERIC, ProQuest, ISI Web of Knowledge, Latin American and Caribbean Health Sciences, Ingenta, and ISI Science Citation Index	To review factors that are positively or negatively associated with ICT adoption by healthcare professionals in clinical settings	101
Gagnon 2016	2016	JAMIA	4	PubMed, Embase, CINHAL, and PsychInfo	To synthesize current knowledge of the factors influencing healthcare professional adoption of mobile health (m-health) applications.	33
Garvey 2022	2022	JMIR Medical Informatics	3	MEDLINE, CINAHL, and the Cochrane Library of Systematic Reviews	To systematically identify research on provider competencies needed for the use of AI in clinical settings	4
Garavand 2022	2022	Informatics in Medicine Unlocked	4	Web of Science, PubMed, Scopus, and Embase	To identify the behavioral factors influencing the acceptance of telemedicine technology among physicians in different contexts	37
Ghimire 2023	2023	Int J Med Inform	4	PubMed, Scopus, Cochrane Library of Systematic Reviews, and Web of Science	To assess the practical implications of virtual prenatal care and identify the needs and experiences associated with it	23
Gonçalves R 2023	2023	J Med Internet Res	7	MEDLINE, Embase, BIREME, IEEE Xplore, BVS, Google Scholar, and Grey literature	To assess evidence on health professionals’ perceptions of the usability of telehealth systems in primary care of individuals with hypertension and diabetes from the COVID-19 pandemic onward	11
Grant 2022	2022	Australian J of Rural Health	7	Scopus, CINAHL, MEDLINE, PEDro, Speechbite, OTseeker and ScienceDirect	To identify the attitudes and perspectives of speech pathologists, occupational therapists and physiotherapists on using telehealth videoconferencing for service delivery to children with developmental delays	14
Hagstram 2022	2022	J Med Internet Res	3	PubMed, CINAHL, and PsycINFO	To identify, categorize, and summarize knowledge about different stakeholders’ (e.g., children and adolescents, parents, HCPs, policy makers, and designers of patient portals or PAEHRs) views, use, and experiences of EHR access for children, adolescents, and parents.	74
Huang 2023	2023	J Med Internet Res	5	PubMed, Scopus, PsycINFO, Embase, and CINAHL	To provide an overview of the research on the use of intelligent physical robots in health care through a systematic literature review, especially to identify its antecedents and consequences	94
Ionescu 2022	2022	JAMIA	8	Embase, MEDLINE, Web of Science Core Collection, WHO GHL, SCIELO, CINAHL EBSCOhost, ERIC Ovid	To create an overview of what is currently known in the literature about the use and implementation of e-consultation and e-learning by HCWs in LMICs and whether there is evidence of complementarity in the joint use of these 2 tools	96
Isidori 2022	2022	JMIR nursing	3	PubMed, Google Scholar, and Web of Science	To review and define the role of nurses and the skills they are asked to master in terms of new methodological approaches and digital knowledge that have emerged before and during the COVID-19 pandemic (2011-2021)	60
Ismatullaev 2022	2022	Human Factors	3	IEEE Xplore, Springer Link and Google Scholar	To provide a comprehensive overview of the factors impacting technology adoption, to predict the acceptance of artificial intelligence (AI)-based technologies	85
Jacob 2020	2020	JMIR mHealth and uHealth	4	MEDLINE, PubMed, Cochrane Library of Systematic Reviews, and the SAGE database	To systematically explore relevant published literature to synthesize the current understanding of the factors impacting clinicians’ adoption of mHealth tools, not only from a technological perspective but also from social and organizational perspectives	171
Jimenez 2020	2020	Int J Med Inform	4	MEDLINE, Embase, CINAHL, and Cochrane Library of Systematic Reviews	To examine the broad literature on DHCs as it applies to Primary Care (PC) settings	28
Jimma 2022	2022	Informatics in medicine unlocked	4	PubMed, Scopus, ProQuest, and Science Direct	To show the best available evidence associated with the obstacles to the acceptance of the electronic medical record system.	21
Joo 2022	2022	Computers, Informatics, Nursing	5	CINAHL, Ovid, PubMed, PsycINFO, and Web of Science	To identify the strengths and weaknesses of nurse-led telehealth interventions for the care of community-dwelling outpatients during the COVID-19 pandemic.	23
Jonasdottir 2022	2022	Int J Med Inform	4	Scopus, PubMed, ProQuest, and EBSCOhost	To answer the research question, “what is known in the literature about challenges and opportunities of telehealth service provision from the perspective of health professionals?“	22
Jose 2023	2023	Int J Environmental Research and Public Health	3	PubMed, Scopus, and Web of Science	To analyse the previous research related to the competence requirements when adopting Healthcare 4.0 technologies	44
Kane 2022	2022	JMIR human factors	3	PubMed, Cairn, Ascodocpsy	To describe the uses of digital technologies at the time of COVID-19 and their impact on professional practices in psychiatry and mental health and to understand the place of digital technologies in the organizational adaptations linked to the COVID-19 epidemic, but also to identify how this specific context questions the modalities of care.	61
K Zhang 2022	2022	J of Interprofessional Care	6	CIPE. PubMed, CINAHL, ERIC, PsycINFO, Cochrane Library of Systematic Reviews, and Google/Google Scholar	To identify the program features and areas of behavior change in healthcare professionals using e-learning	32
Keyworth 2018	2018	BMC Med Inform Decis Mak	6	MEDLINE, Embase, CINAHL, PsycINFO, Web of Science, and Cochrane Library of Systematic Reviews	To identify interventions with a technological component that are successful at changing professional practice, to determine if and how such interventions are theory-based, and to examine barriers and facilitators to successful implementation	69
Koivunen 2018	2018	Scand J Caring Sci	6	PubMed/MEDLINE, CINAHL, ProQuest, Web of Science, Scopus, Finnish Medic, and Ohtanen	To synthesize the best available research evidence on nursing professionals’ experiences of the facilitators and barriers to the use of online telehealth services in nursing practice	25
Kolla 2021	2021	J Public Health Manage Pract	2	PubMed and Google Scholar	To conduct a scoping review on health informatics-based strategies for CHW-provider communication that aim to improve integration of CHWs into clinical settings; discuss their advantages, limitations, and future directions to maximize these strategies in the context of clinical care	31
Konnyu 2023	2023	Obstetrics and gynecology	6	Medline (through PubMed), the Cochrane Register of Clinical T1ials, the Cochrane Database of Systematic Reviews, EMBASE, CINAHL, and Clinica!Ttials.gov	To systematically review patient, partner or family, and clinician perspectives, preferences, and experiences related to prenatal care visit schedules and televisits for routine prenatal care	9
Kruse 2022	2022	J Med Internet Res	4	PubMed, CINAHL, Web of Science, and ScienceDirect	To examine physician burnout issues incident to the EHR prior to and during the first year of the COVID-19 pandemic by analyzing the literature from the last 5 years	25
Laar 2022	2022	BMC Health Services Research	6	Medline, Scopus, PsychINFO, CINAHL and Cochrane Library, Google	To identify HCPs perspectives on barriers to, and facilitators of, mobile phone based SRH services and information in rural areas of LMICs from current literature.	12
Lam 2022	2022	npj Nature	4	MEDLINE, Embase, Web of Science, and IEEE Xplore	To systematically review the literature and determine the ML techniques used for technical surgical skill assessment and identify challenges and barriers in the field	66
Lampickien˙e 2022	2022	Life (Basel, Switzerland)	3	PubMed, Web of Science, and IEEE Xplore	To explore the existing literature concerning the user experience of digital care visits (telemedicine) from different healthcare professionals’ points of view	28
Li 2013	2013	Interac J Med Res	8	MEDLINE, Cinahl, Web of Science, PubMed, PsychInfo, ERIC, ProQuest Science Journals, and Embase.	To identify and synthesize the factors influential to health care providers’ acceptance of various eHealth applications.	93
Li 2019	2019	Telemedicine. e-Health	11	Cochrane Library of Systematic Reviews, Academic Search Premier, CINAHL, British Education Index, CDAS, CMMC, EA, LISTA, MEDLINE, MLA International Bibliography, and Web of Science	To critique and summarize existing research on ICU nurses’ perspectives toward the telemedicine intensive care unit (Tele-ICU). In addition to this, find evidence to support implementation of Tele-ICU program in China	14
Lluch 2011	2011	Intl J Med Inform	25	Cochrane Library of Systematic Reviews, CSA Illumina, EBSCOHOST, JSTOR, Collections, ACM, ProQuest, Emerald Journals, Ingenta, PubMed, Science Direct, Google Scholar, EPPI Centre, CDR, Rand Corportation, Joanna Briggs Institute, NICE, SCIE, The Commonwealth Fund, CHSRF, Government sources, and think tanks	To identify and categorize, from an organizational management perspective, barriers to use of or ICT adoption for health and future policy interventions	79
Longhini 2022	2022	J Med Internet Res	4	MEDLINE, CINAHL, PsycINFO, and Scopus	To summarize digital health competencies investigated to date and the tools used to assess them among health care professionals	26
Martin 2019	2019	JAMIA	7	MEDLINE, PsycINFO, Embase, CINAHL Plus, HMIC, Cochrane Library of Systematic Reviews, and National Institute of Health Research HTA	To summarize the quality and breadth of evidence for the impact of mobile technologies on communication and teamwork in hospitals	30
Marvaso 2022	2022	Applied Sciences (Switzerland)	4	Dimensions database, Embase, PubMed, and Web of Science	To provide a glance at the recent developments in augmented reality/virtual reality to support students’ education, personnel training and patients’ empowerment in this clinical setting	41
Meunier 2023	2023	Annals of Family Medicine	5	PubMed, PsycInfo, Embase, CINAHL, and Cochrane Library of Systematic Reviews	To identify and quantify the barriers and facilitators to the use of CDSSs by primary care professionals	48
Moore 2020	2020	JAMIA	6	Embase, MEDLINE, CINAHL, Scopus, PsycInfo, and Web of Science	To study the impact of health information technology on nurses’ time and to address the knowledge gap	33
Muhiyaddin 2020	2020	Stud Health Technol Inform	3	CINAHL, PubMed, and Google Scholar	To explore the impact of the CDSS on physicians as reported through the literature	14
Mulita 2022	2022	Sensors (Basel, Switzerland)	2	PubMed and Web of Science	To summarize the most important studies evaluating the internet of things concept within surgical practice, focusing on Telesurgery and surgical telementoring	48
Namasivayam 2022	2022	PloS one	7	MEDLINE, CINAHL, Scopus, Web of Science Core Collection, Embase, PsycINFO, and Emcare	To review and map the available evidence on the use of telehealth in providing after-hours palliative care services in rural and remote Australia.	12
Nezamdoust 2022	2022	Journal of Research in Nursing: JRN	6	Google Scholar, Scopus, Cochrane Library of Systematic Reviews, Embase, Ovid, and PubMed	To study the utilization of mobile health applications by nurses and presenting a scenario of how and why they are utilized	25
Nguyen 2021	2021	JAMIA	6	MEDLINE, Embase, CINAHL, PsycINFO, ProQuest, and Web of Science	To assess the multilevel (organizational, physician, and information technology [IT]) factors associated with EHR-related impacts on physician well-being and burnout and to identify promising potential EHR improvements, as recommended by physicians	35
Niazkhani 2020	2020	BMC Med Inform Decis Mak	6	MEDLINE, PubMed, Science Direct, CINAHL, CENTRAL, and IEEE	To identify the types of barriers to a patient, provider, and caregiver adoption/use of ePHRs and to analyze their extent in chronic disease care	60
Nizeyimana 2022	2022	Digital health	7	PubMed, Scopus, PEDro, Cochrane library, EBSCOhost (Academic search premier, Africa-wide information, CINAHL, Eric, MEDLINE, Health sources - Nursing/Academic edition), Africa online, and ProQuest databases	To scope all published information reporting on the feasibility, cost, access to rehabilitation services, implementation processes including barriers and facilitators of telerehabilitation (TR) in low- and middle-income countries (LMICs) and high-income countries (HICs).	29
O’Connor 2022	2022	Journal of Clinical Nursing	4	CINAHL, Embase, PubMed, and Scopus	To identify and summarize the scientific literature on AI in nursing and midwifery, to identify the extent of nurses and midwives’ involvement in the development, delivery, or use of AI in healthcare, to identify methods AI being employed across the nursing and midwifery professions in terms of clinical practice, education, research, and policy, to identify the benefits, limitations, and risks of AI in nursing and midwifery?	140
Odendaal 2020	2020	Cochrane Database of Syst Rev	13	MEDLINE, Embase, CINAHL, SSCI, Global health, Eldis, Google Scholar, mHealth database, mHealth Evidence, mHealth Knowledge, mPowering, OpenGrey, and Grey Literature	To synthesize qualitative research evidence on health workers’ perceptions and experiences of using mHealth technologies to deliver primary healthcare services, and to develop hypotheses about why some technologies are more effective than others	53
Osman 2019	2019	BMC Glob Health	8	MEDLINE, Embase, Cochrane Library of Systematic Reviews, CINAHL, PsycINFO, ProQuest, Conference Proceedings Citation Index, and Google search	To investigate factors (barriers and facilitators) influencing the adoption and implementation of electronic consultation (eConsult services) to enhance access to speacialist care	130
Papadopoulos 2018	2018	Contemporary Nurse	7	MEDLINE, PubMed, CINHAL, Embase, PsycInfo, Web of Science, and IEEE Xplore	To provide an overview of the existing evidence related to the views of nurses and other health and social care workers about the use of assistive humanoid and animal-like robots	19
Police 2011	2011	Inform Prim Care	3	MEDLINE, Embase, and Grey Literature	To better understand current utilization rates along with benefits and barriers to HIT adoption in physician practice organizations	119
Prakash 2022	2022	J of Personalized Medicine	2	PubMed and Google Scholar	To scrutinize the ethical complications associated with the application of artificial intelligence in the healthcare field	16
Rahal 2021	2021	BMC Med Inform Decis Mak	4	MEDLINE, PsycINFO, Embase, and PROSPERO	To explore and identify the factors that impact Primary Care Physcians’ mature use of EMR	14
Ramachandran 2023	2023	Heart and Lung	9	PubMed, Embase, CINAHL, Cochrane Library of Systematic Reviews, Scopus, PsycINFO, Web of Science, ERIC, and Proquest Dissertations and Theses Global	To summarize patient- and healthcare provider-level barriers and facil- itators in the adoption of DHIs for COPD management	27
Ratshidi 2022	2022	Sustainability	7	Scopus, PubMed, Web of Science, Science direct, Google, Google scholar, CHW Central website	To conceptualise the social factors to consider when implementing a bespoke ICT solution suited to the specific demands of CHWs in primary healthcare in developing contexts, with a particular focus on the South African context	59
Rukavina 2021	2021	J. Med. Internet Res.	3	PubMed, CINAHL, and Scopus	The purpose of this scoping review is to characterize the recent original peer-reviewed research studies on the e-professionalism of HCPs; to assess the quality of the methodologies and approaches used; to explore the impact of SM on e-professionalism of HCPs; to recognize the benefits and dangers of SM; and to provide insights to guide future research in this area	88
Saigí-Rubió 2022	2022	J Med Internet Res	5	PubMed, Embase, Web of Science, Cochrane Library of Systematic Reviews, and Scopus	To summarize findings regarding the use of telemedicine across the 53 member states of the WHO European Region and to identify the medical fields and levels of care in and at which the effectiveness, feasibility, and applicability of telemedicine have been demonstrated	33
Sipanoun 2022	2022	Int J of Med Inform	8	Embase, EMCARE, MEDLINE, Cochrane Library, Web of Science, Scopus, CINAHL and PsycINFO	To understand the experiences and perceptions of all relevant stakeholders using an EMR system in the pediatric hospital setting, including the use of an EMR-linked patient portal	36
Sullivan 2022	2022	Social Work in Public Health	5	Google Scholar, VA Library Network, JSTOR, PubMed, ERIC via EBSCOhost	To assess the current responses from the field of social work during the COVID-19 pandemic, leveraging telemedicine, social work, self-care, and the fluidity of VA services	10
Tabaeeian 2022	2022	J of Science and Technology Policy Management	2	Scopus and PubMed	To identify barriers to the use of telemedicine systems in primary health-care individual level among professionals	37
Tegegne 2023	2023	Interactive J of Medical Research	6	PubMed, Web of science, African journals OnLine, EMBASE, Medline, Scopus	To determine the pooled estimate of EMR use and success determinants among health professionals in Ethiopia.	5
Tickner 2023	2023	Social work in health care	7	CINAHL, Embase, Medline, PsycINFO, Scopus, Social Work Abstracts and Sociological Source Ultimate	To explore what is known about the use of eHealth technologies in health social work practice	25
Torres-Castaño 2023	2023	Int J Environmental Research and Public Health	2	MEDLINE and Embase	To identify the impact of the ELSI dimensions and other dimensions, such as the organizational and environmental, to analyze in depth the challenges of the implementation of teleneurology as a complement to face-to-face neurology care	53
Poissant 2005	2005	JAMIA	4	MEDLINE, CINAHL, HealthSTAR, and Current Health	To estimate the extent to which an Electronic Health Records affects clinicians’ documentation time and to identify factors that may explain efficiency differences observed across studies	23
Thomas-Craig 2021	2021	JAMIA	4	MEDLINE, Embase, Cochrane Library of Systematic Reviews, and ACM	To identify and summarize interventions used to address the burden of digital tools and their impact on workflow inefficiencies	81
Vejdani 2022	2022	BMC Med Inform Decis Mak	4	Web of Science, PubMed, Scopus, and ProQuest	To identify the requirements of the electronic prescribing system	13
Verma 2022	2022	BJGP Open	2	PubMed and PsychInfo	To synthesize data on patients’ and PCPs’ experiences with remote consultations in the primary care setting to inform future research and policy in this area	24
von Wedel 2020	2020	J Med Internet Res	2	PubMed and Google Scholar	To provide a comprehensive overview including a variety of technologies beyond computer-based patient records	50
Walle 2023	2023	Informatics in Medicine Unlocked	8	MedLine, PubMed, Scopus, EMBASE, African Journal Online, HINARI, Science Direct, Web of science	To identify the pooled levels of readiness to use EMRs and associated factors among health professionals in Ethiopia	3
Walsh 2021	2021	Clinical Ophthalmology	5	Embase, PubMed, Web of Science, Google Scholar, Google	To conduct a systematic review identifying, describing and contrasting teleophthalmology services in NZ with the comparable countries of Australia, USA, Canada and the United Kingdom	132
Wisner 2019	2019	Int J Nurs Stud	5	MEDLINE/PubMed, CINAHL, Embase, Web of Science, and PsycINFO	To synthesize the literature on the electronic health record’s impact on nurses’ cognitive work	18
Xyrichis 2021	2021	Cochrane Database of Syst Rev	4	MEDLINE, Embase, CINAHL, and Web of Science	To identify, appraise and synthesize qualitative research evidence on healthcare stakeholders’ perceptions and experiences of factors affecting the implementation of CCT, and to identify factors that are more likely to ensure successful implementation of CCT for subsequent consideration and assessment in telemedicine effectiveness reviews	13
Young 2011	2011	Chest	5	PubMed, CINAHL, Global Health, Web of Science, and Cochrane Library of Systematic Reviews	To systematically evaluate the published and unpublished literature addressing the acceptance of tele-ICU coverage by ICU staff with a focus on benefits and challenges seen by frontline providers in adopting this new technology	23
Zakerabasali 2021	2021	Healthc Informatics Res	4	PubMed, Embase, Web of Science, and Google Scholar	To conduct a systematic review of more recent literature on barriers associated with mHealth reported by healthcare professionals and identify the most important barriers	18
Zhang J 2023	2023	Int Orthopaedics	3	PubMed MEDLINE, Ovid EMBASE and Scopus.	To identify the type of XR most frequently used in various surgical specialties and phases of surgical intervention, identify key outcome measures and trends for the use of XR in surgery, determine if XR has been a promising addition to surgery, and which aspect of surgical practice has benefited the most, and to identify opportunities and challenges for XR development and usage in the future	168
Zhang Z 2023	2023	JMIR Medical Informatics	6	ACM Digital Library, Cochrane Library, IEEE Xplore, Ovid MEDLINE, Embase, and Web of Science	To synthesize the knowledge and experiences of smart glasses, understand the benefits and limitations regarding adopting smart glasses as a telemedicine tool, and inform the design of future smart glass applications to better support remote care coordination	21
**Protocols registered and identified**				**Protocol objective**		
Abreu 2018	2018	PROSPERO	N/A	To understand the use and impact of mHealth by community health workers in developing and least developed countries		N/A
Bajgain 2023	2023	BMJ open	6	To map and synthesize determinants (barriers and facilitators) to implementing AI-based CDS tools in healthcare.		N/A
Cherifi 2021	2021	PROSPERO	N/A	To evaluate what are the barriers and enablers to implementing TD with dental healthcare professionals		N/A
Jacques 2019	2019	PROSPERO	N/A	To answer the question: “Does the scientific evidence available in the literature demonstrate the effectiveness of the use of web applications to promote the mental health of health workers?”		N/A
Luangphituck 2023	2023	JMIR research protocols	N/A	To synthesize the best available evidence concerning the preventive effect of internet-based cognitive behavior therapy on employees		N/A
Mahmood 2018	2018	PROSPERO	N/A	To answer the question: “What are the various community health worker-based mobile health approaches to improve the management and knowledge/perception of caregivers regarding common childhood infections?”		N/A
Mbuthia 2018	2018	PROSPERO	N/A	To understand how m-Health communication strengthen postnatal care in rural areas		N/A
Park 2020	2020	PROSPERO	N/A	To analyze how effective is m-Health intervention in reducing the burden of caregivers of dementia patients		N/A
Wootton 2011	2011	BMC Health Ser. Res.	N/A	Estimate the travel reduction associated with the use of telemedicine by patients and healthcare professionals		N/A

*ACM* Association for Computing Machinery, *AHRQ* Agency for Healthcare Research and Quality, *AMED* Allied and Complementary Medicine Database, *ASSIA* Applied Social Sciences Index and Abstracts, *CCT* Critical Care Telemedicine, *CDAS* Child Development & Adolescent Studies, *CDR* York Centre for Reviews and Dissemination, *CDSS* Clinical Decision Support System, *CENTRAL* Cochrane Central Register of Controlled Trials, *CHF* Chronic Heart Failure, *CHSRF* Canadian Health Services Research Foundation, *CHW* Community Health Worker, *CINAHL* Cumulative Index to Nursing and Allied Health Literature, *CMMC* Communication & Mass Media Complete, *COPD* Chronic Obstructive Pulmonary Disease, *DARE* Database of Abstract of Reviews of Effectiveness, *EA* Education Abstracts [H.W. Wilson], *EHI* Electronic Health Information, *EMR* Electronic medical record, *HCP* Health Care Professionals, *HCW* Health Care Worker, *HMIC* Healthcare Management Information Consortium, *HSRProj* Health Services Research Projects in Progress, *HSTAT* Health Services, Technology, Assessment Text, *HTA* Health Technology Assessment, *ICT* Information Communication Technology, *ICTRP* International Clinical Trials Registry Platform, *ICU* Intensive Care Unit, *INSPEC* Database for Engineering Researchers, *LISA* Library and Information Science Abstracts, *LISTA* Library, Information Science, & Technology Abstracts, *LMIC* Low- and middle-income countries, *NHS* National Health System, *NICE* National Institute for Health and Clinical Excellence, *PopLine Database* Database from the University of London, *RMT* Remote Monitoring Technology, *SCIE* Social Care Institute for Excellence, *SNS* Social Network Sites, *SSCIE* Social Sciences Citation Index Expanded, *SSSCI* Science and Social Science Citation Indices, *TD* Teledentistry, *UK* United Kingdom, *VC* Video Consultations, *WHO* World Health Organization.

*In the abstract of the referred review it is stated the inclusion of 15 databases; however, there is a description of only 13 databases (value considered correct).

**Table 2:** Population being evaluated, studies’ methodologies and technologies being evaluated.

Study ID	Targeted health workers	Number of health workers	Study Designs Included	Technology under investigation	Geographic region	Disease or Condition Considered (Based on the ICD-10)
Addotey-Delove 2023	Healthcare professionals in general	N/A	Quantitative studies	1. m-Health technologies	African, Asian, and Latin American regions	N/A
Adepoju 2017	Community health workers, nurses, clinicians, clinical officers, and healthcare professionals in general	N/A	Qualitative, quantitative, and mixed qualitative-quantitative studies	1. CDSS	African region	Maternal and prenatal health, childhood illness, tuberculosis, HIV, and Hypertension
Alkhaldi 2023	Healthcare professionals in general	N/A	Qualitative, quantitative, and mixed qualitative-quantitative studies	1. m-Health technologies	European and North American regions, and Australia	N/A
Al-Rawashdeh 2022	Healthcare professionals in general	N/A	Qualitative, quantitative, and mixed qualitative-quantitative studies	1. Internet of things	Asian, European, and Latin American regions	N/A
Agarwal 2015	Frontline health workers, midwives, nurses, and outpatient health care workers	N/A	Qualitative studies	1. m-Health technologies	African, Asian, and Latin American regions	Anemia, tuberculosis, drug-dosing, pre- and post-natal care, family planning, postpartum hemorrhage, and HIV
Amoakoh-Coleman 2016	Healthcare professionals in general, community health workers, health surveillance assistants, and midwives	N/A	Qualitative and quantitative studies	1. m-Health technologies	African and Asian regions	Maternal health, HIV, post-natal depression, and malaria in pregnancy
Arsad 2023	General practitioners and healthcare professionals in general	1130	Qualitative, quantitative, and mixed qualitative-quantitative studies	1. eHealth technologies	Asian and European regions	N/A
Aslani 2022	Physicians and nurses	N/A	Quantitative studies	1. Telehealth, telemedicine, telemonitoring, and remote monitoring technologies	Asian, European, and Latin and North American regions	Cardiovascular diseases
Avoka 2022	Healthcare professionals in general	N/A	Qualitative, quantitative, and mixed qualitative-quantitative studies	1. m-Health technologies	African region	Maternal health
Baluszek 2022	Healthcare professionals in general	N/A	Qualitative and mixed methods study	1. Telehealth, telemedicine, telemonitoring and remote monitoring technologies	European region	N/A
Bervell 2019	Healthcare professionals in general	N/A	Quantitative, qualitative, and mixed qualitative-quantitative studies	1. m-Health technologies 2. Electronic medical records and clinical information systems 3. Telehealth, telemedicine, telemonitoring, and remote monitoring technologies 4. e-Health technologies	African region	Infectious, cardiovascular, and oral diseases
Boonstra 2010	Physicians	25624	Quantitative, qualitative, and mixed qualitative-quantitative studies	1. Electronic medical records and clinical information systems	European region	N/A
Brommeyer 2023	Healthcare professionals in general	N/A	Qualitative and quantitative studies	1. Electronic medical records and clinical information systems	Asian, European, and Latin and North American regions, and Australia	N/A
Braun 2013	Community health workers	N/A	Qualitative and quantitative studies	1. m-Health technologies	African, Asian, and Latin American regions	Sexual, reproductive, maternal illnesses, child health, HIV, tuberculosis, and malaria
Brewster 2014	Front-line professionals	228	Quantitative, qualitative, and mixed qualitative-quantitative studies	1. Telehealth, telemedicine, telemonitoring, and remote monitoring technologies	European region and Australia	COPD and CHF
Brown 2020	Nurses	41176	Quantitative, qualitative, and mixed qualitative-quantitative studies	1. Electronic medical records and clinical information systems 2. m-Health technologies	African, Asian, European, and Latin and North American regions, and Australia	N/A
Calleja 2022	Healthcare professionals in general	N/A	Qualitative, quantitative, and mixed qualitative-quantitative studies	1. Telehealth, telemedicine, telemonitoring, and remote monitoring technologies	African, Asian, European, Latin and North American regions, and Australia	N/A
Cansdale 2022	Nurses, birth attendants, and community health workers	1486	Qualitative, quantitative, and mixed qualitative-quantitative studies	1. m-Health technologies	African, Asian, and Latin American regions	Neonatology
Cartolovni 2022	Healthcare professionals in general	N/A	Original research	1. eHealth technologies	Asian, European, and North American regions, and Australia	N/A
Celes 2018	Healthcare profesionals in general	N/A	Quantitative studies	1. Telehealth, telemedicine, telemonitoring and remote monitoring technologies	African, Asian, European, Latin and North American regions, and Australia	N/A
Cen 2022	Pharmacists	N/A	Quantitative and qualitative studies	1. eHealth technologies	Asian, European, and North American regions, and Australia	N/A
Chan 2018	Healthcare professionals in general	N/A	Quantitative, qualitative, and mixed qualitative-quantitative studies	1. Social media network platforms	N/A	N/A
Chen 2022	Physicians and healthcare professionals in general	14049*	Qualitative, quantitative, and mixed qualitative-quantitative studies	1. Artificial intelligence	Asian, European, Latin and North American regions, and Australia and New Zealand	N/A
Christensen 2020	Mental health practitioners	N/A	Quantitative and qualitative studies	1. Telehealth, telemedicine, telemonitoring, and remote monitoring technologies	Asian and European regions, and Australia	Unipolar depression
Da Costa 2020	Dental health services providers	N/A	Quantitative, qualitative, mixed qualitative-quantitative studies, and economic analysis	1. Telehealth, telemedicine, telemonitoring, and remote monitoring technologies	Asian, European, and Latin and North America regions, and Australia	Dental health conditions
Davis 2014	Primary care professionals, medical assistants, clinicians, consultants, and healthcare professionals in general	N/A	Quantitative, qualitative, and mixed qualitative-quantitative studies	1. Telehealth, telemedicine, telemonitoring, and remote monitoring technologies	Asian, European, and North American regions	Diabetes, cardiac diseases, lung diseases, and cancers
de Grood 2016	Physicians	N/A	Quantitative, qualitative, and mixed qualitative-quantitative studies	1. Electronic medical records and clinical information systems 2. Telehealth, telemedicine, telemonitoring, and remote monitoring technologies	North American region	N/A
Drissi 2021	Healthcare professionals in general	N/A	N/A	1. Social media network platforms 2. Telehealth, telemedicine, telemonitoring, and remote monitoring technologies 3. m-Health technologies	Asian, European, and North American regions	Post-traumatic stress disorder, anxiety, and stress
Dutta 2020	Physicians	N/A	N/A	1. Electronic medical records and clinical information systems	African, Asian, and North American regions	N/A
Early 2019	Community health workers	N/A	Quantitative, qualitative, and mixed qualitative-quantitative studies	1. m-Health technologies	African and Latin and North American regions, and Australia	Maternal, child, and reproductive health, tuberculosis, and HIV
Ebneter 2022	Healthcare professionals in general	N/A	Quantitative, qualitative, and mixed qualitative-quantitative studies	1. Telehealth, telemedicine, telemonitoring, and remote monitoring technologies	European region	Palliative care
Emmett 2022	Healthcare professionals in general	111	Qualitative and mixed qualitative-quantitative studies studies	1. m-Health technologies	Australia	Cardiovascular diseases
Ferdousi 2021	Nurses	3989	Quantitative and qualitative studies	1. Electronic medical records and clinical information systems	Asian region	N/A
Fletcher 2023	Healthcare professionals in general	N/A	Qualitative, quantitative, and mixed qualitative-quantitative studies	1. CDSS	African, Asian, European, North American regions, and Australia	Miscellaneous (oncology, cardiology, infectious diseases, and others)
Ftouni 2022	Healthcare professionals in general	N/A	Quantitative and qualitative studies	1.Telehealth, telemedicine, telemonitoring and remote monitoring technologies	African, Asian, European, Latin and North American regions	N/A
Gagnon 2012	Healthcare professionals in general	N/A	Quantitative, qualitative, and mixed qualitative-quantitative studies	1. Electronic medical records and clinical information systems 2. m-Health technologies 3. Telehealth, telemedicine, telemonitoring, and remote monitoring technologies 4. CDSS 5. Clinical reminder and alert systems 6. Laboratory reporting system 7. Personal Digital Assistant 8. Clinical information systems 9. E-learning	European and North American regions, and Australia	N/A
Gagnon 2016	Healthcare professionals in general	N/A	Quantitative, qualitative, and mixed qualitative-quantitative studies	1. m-Health technologies 2. Telehealth, telemedicine, telemonitoring, and remote monitoring technologies	African, Asian, and European regions, and Australia	N/A
Garavand 2022	Physicians	N/A	Qualitative, quantitative, and mixed qualitative-quantitative studies	1. Telemedicine, telehealth, telemonitoring and remote monitoring technologies	African, Asian, European, and North and Latin American regions	N/A
Garvey 2022	Healthcare professionals in general	22	Quantitative studies	1. Artificial intelligence	North American region	N/A
Ghimire 2023	Healthcare professionals in general	51	Quantitative and Qualitative	1. Telemedicine, telehealth, telemonitoring and remote monitoring technologies	Asian, European, and North American regions	Pregnancy and maternal health
Gonçalves R 2023	Healthcare professionals in general	248	Quantitative and qualitative studies	1. Telemedicine, telehealth, telemonitoring and remote monitoring technologies	Asian, European, and Latin and North American regions, and Australia	Chronic diseases (including DM and hypertension)
Grant 2022	Speech pathologists, Occupational therapists and Physiotherapists	N/A	Quantitative, Qualitative, and mixed qualitative-quantitative studies	Telemedicine, telehealth, telemonitoring and remote monitoring technologies	North American and Australian region	Children with Developmental delays
Hagstram 2022	Healthcare professionals in general	496	Qualitative, quantitative, and mixed qualitative-quantitative studies	1. Electronic medical records and clinical information systems	European and North American regions, and Australia	Pediatrics
Huang 2023	Healthcare professionals in general	N/A	Qualitative, quantitative, and mixed qualitative-quantitative studies	1. Intelligent Physical Robots	N/A	N/A
Ionescu 2022	Healthcare professionals in general	N/A	Qualitative, quantitative, and mixed qualitative-quantitative studies	1. E-learning 2. Telehealth, telemedicine, telemonitoring, and remote monitoring technologies 3. Electronic medical records and clinical information systems	African, Asian, and Latin American regions	Maternal health, infectious diseases, such as HIV/AIDs, and tuberculosis
Isidori 2022	Nurses	N/A	Quantitative, qualitative, and mixed qualitative-quantitative studies	1. Telehealth, telemedicine, telemonitoring, and remote monitoring technologies	N/A	N/A
Ismatullaev 2022	Healthcare professionals in general	N/A	N/A	1. Artificial intelligence	N/A	N/A
Jacob 2020	Physicians	N/A	Quantitative, qualitative, and mixed qualitative-quantitative studies	1. mHealth technologies	African, Asian, European, North and Latin America, and Australia and New Zealand	Miscellaneous (acute diseases, diabetes, mental disorders, and others)
Jimenez 2020	Primary healthcare professionals	N/A	Quantitative, qualitative, and mixed qualitative-quantitative studies	1.Electronic medical records and clinical information systems 2.Telehealth, telemedicine, telemonitoring and remote monitoring technologies 3. mHealth technologies 4. Personal Digital assisstant	African, European, North American regions, and Australian region	N/A
Jimma 2022	Physicians and nurses	N/A	Quantitative, qualitative, and mixed qualitative-quantitative studies	1. Electronic medical records and clinical ainformation systems	African, Asian, European and North American regions	N/A
Joo 2022	Nurses	N/A	Quantitative, qualitative, and mixed qualitative-quantitative studies	1. Telehealth, telemedicine, telemonitoring, and remote monitoring technologies	Asian, European, and North American regions, and Australia and New Zealand	Cardiovascular disease and oncology
Jonasdottir 2022	Healthcare professionals in general	N/A	Qualitative, Quantitative and mixed qualitative - quantitative studies	1. Telehealth, telemedicine, telemonitoring and remote monitoring technologies	African, Asian, European, North American regions, and Australia	N/A
Jose 2023	Healthcare professionals in general	N/A	Quantitative and qualitative studies	1. mHealth technologies 2. Telehealth, telemedicine, telemonitoring and remote monitoring technologies 3. Electronics medical records and clinical information systems	N/A	N/A
Kane 2022	Health care professionals, Psychiatrist, Community Health	N/A	Qualitative and Quantitative studies	1. Telehealth, telemedicine, telemonitoring and remote monitoring technologies 2. mHealth technologies 3. Social media network platforms 4. Artificial intelligence	African, Asian, European, North American regions, and Australian region	Psychiatry and Mental health
K. Zhang 2022	Healthcare professionals in general	35542	Qualitative, Quantitative and mixed qualitative - quantitative studies	1. E-Learning 2. Telehealth, telemedicine, telemonitoring, and remote monitoring technologies 3. Social media network platforms 4. mHealth technologies	North American region and Australian region	N/A
Keyworth 2018	Healthcare professionals in general	N/A	Quantitative, qualitative, mixed qualitative-quantitative studies	1. e-Health technologies	European and North American regions, and Australia	N/A
Koivunen 2018	Healthcare professionals in general	364	Qualitative and mixed methods studies	1. Telehealth, telemedicine, telemonitoring, and remote monitoring technologies 2. m-Health technologies	N/A	N/A
Kolla 2021	Community health workers	N/A	Quantitative, qualitative, mixed qualitative-quantitative studies	1. Electronic medical records and clinical information systems 2. m-Health technologies 3. Cloud- and web-based systems	North American region	N/A
Konnyu 2023	Healthcare professionals in general	674	Qualitative studies	Telehealth, telemedicine, telemonitoring and remote monitoring technologies	European and North American region	N/A
Kruse 2022	Physicians	N/A	Quantitative, qualitative, and mixed qualitative-quantitative studies	1. Electronic medical record and clinical information systems	N/A	Mental Health
Laar 2022	Health workers, Community health workers, Health care providers in general, Nurses	N/A	Qualitative studies	1. mHealth technologies	Asian regions	Sexual and reproductive health
Lam 2022	Surgeons	1603	Quantitative studies	1. Artificial intelligence	African, Asian, European, North American regions, and Australia	N/A
Lampickienė 2022	Mental health professionals, physicians, surgeons, Nurses	N/A	Quantitative, qualitative, mixed qualitative-quantitative studies, and review	1. Telehealth, telemedicine, telemonitoring, and remote monitoring technologies	European and North American regions, and Australia	N/A
Li 2013	Healthcare professionals in general	N/A	Qualitative and quantitative studies	1. Telehealth, telemedicine, telemonitoring, and remote monitoring technologies 2. Electronic medical records and clinical information systems	Australia	N/A
Li 2019	Nurses	2106	Qualitative and quantitative studies	1. Telehealth, telemedicine, telemonitoring, and remote monitoring technologies	Asian region	Critical care conditions
Lluch 2011	Healthcare professionals in general	N/A	Quantitative, qualitative, mixed qualitative-quantitative studies	1. Telehealth, telemedicine, telemonitoring, and remote monitoring technologies 2. Electronic medical records and clinical information systems	N/A	N/A
Longhini 2022	Healthcare professionals in general	17143	Quantitative, qualitative, mixed qualitative-quantitative studies	1. e-Health technologies	African, Asian, European, and North American regions	N/A
Martin 2019	Physicians and nurses	> 3705	Qualitative and quantitative studies	1. m-Health technologies	Asian, European, and North American regions, and Australia/New Zealand	N/A
Marvaso 2022	Radiotherapy, RT students, Medical Physics	N/A	Survey	1. Virtual reality or augmentative Reality	N/A	Radiotherapy
Meunier 2023	Primary Care physicians and nurses	59	Quantitative, qualitative, and mixed qualitative-quantitative studies	1. CDSS	African, Asian, European, and Latin and North American regions, and Australia	N/A
Moore 2020	Nurses	N/A	Qualitative and quantitative studies	1. Electronic medical records and clinical information systems	Asian, European, and Latin and North American regions, and Australia	N/A
Muhiyaddin 2020	Physicians	N/A	N/A	1. CDSS	N/A	N/A
Mulita 2022	Healthcare professionals in general	757	Quantitative studies	1. Telehealth, telemedicine, telemonitoring, and remote monitoring technologies	N/A	Surgical field
Namasivayam 2022	Healthcare professionals in general	46	Quantitative, qualitative, and mixed qualitative-quantitative studies	1. Telehealth, telemedicine, telemonitoring, and remote monitoring technologies	Australian region	Palliative care
Nezamdoust 2022	Nurses	N/A	N/A	1. Telehealth, telemedicine, telemonitoring, and remote monitoring technologies	African, Asian, European, Latin and North American regions, and Australia	N/A
Nguyen 2021	Physicians	30182	Quantitative, qualitative, mixed qualitative-quantitative studies	1. Electronic medical records and clinical information systems	North American region	N/A
Niazkhani 2020	Healthcare professionals in general		Quantitative, qualitative, mixed qualitative-quantitative studies	1. Electronic medical records and clinical information systems	European and Latin and North American regions, and Australia/New Zealand	Chronic conditions (such as diabetes, cystic fibrosis, arthritis, hypertension, multiple sclerosis, asthma, and CHF)
Nizeyimana 2022	Health professionals in general	N/A	Quantitative studies	1. Telehealth, telemedicine, telemonitoring, and remote monitoring technologies	African, Asian, European, North American regions, and Australia	N/A
O’Connor 2022	Nurses, and midwives	N/A	Quantitative, qualitative, and mixed qualitative-quantitative studies	1. CDSS 2. Artificial intelligence	Asian, European and Latin and North American regions, and Australia	Maternal and child Health, mental diseases
Odendaal 2020	Healthcare professionals in general	N/A	Quantitative, qualitative, and mixed qualitative-quantitative studies	1. m-Health technologies	African, Asian, European, and Latin and North American regions, and Australia	NA
Osman 2019	Physicians	82420	Quantitative, qualitative, and mixed qualitative-quantitative studies	1. Telehealth, telemedicine, telemonitoring, and remote monitoring technologies	European and Latin and North American regions	N/A
Papadopoulos 2018	Nurses and social care workers	>1545	Quantitative, qualitative, and mixed qualitative-quantitative studies	1. Assistive humanoid and animal-like robots	Asian, European, and North American regions, and Australia and New Zealand	Mostly neurological conditions (including dementia)
Police 2011	Physician	28217	Quantitative, qualitative, and mixed qualitative-quantitative studies	1. Electronic medical record and clinical information systems	North American region	N/A
Poissant 2005	Nurses and physicians	328	Quantitative and qualitative studies	1. Electronic medical records and clinical information systems	Countries were not reported	N/A
Prakash 2022	Healthcare professionals in general	N/A	Quantitative, qualitative, and mixed qualitative-quantitative studies	1. Artificial intelligence	N/A	N/A
Rahal 2021	Physicians	106876	Quantitative and qualitative studies	1. Electronic medical records and clinical information systems	European and North American regions, and Australia/New Zealand	N/A
Ramachandran 2023	Healthcare professionals in general and patients	390	Quantitative, qualitative, and mixed qualitative-quantitative studies	1. Telehealth, telemedicine, telemonitoring, and remote monitoring technologies	European region	COPD
Ratshidi 2022	Community healthcare professionals	N/A	Quantitative, Qualitative and mixed qualitative-quantitative studies	1. mHealth technologies	Asian, African, Latin and North American region	N/A
Rukavina 2021	Healthcare professionals in general	98	N/A	1. Social media network platforms	N/A	N/A
Saigí-Rubió 2022	Healthcare professionals in general	N/A	Quantitative, qualitative, and mixed qualitative-quantitative studies	1. Telehealth, telemedicine, telemonitoring, and remote monitoring technologies	European Region	N/A
Sipanoun 2022	Overall users, including health professionals	1638	Quantitative, qualitative, and mixed qualitative-quantitative studies	1. Electronic medical records and clinical information systems	Asian, European, and North American regions, and Australia	Pediatrics
Sullivan 2022	Healthcare professionals in general	N/A	Qualitative and quantitative methods	1. Telehealth, telemedicine, telemonitoring, and remote monitoring technologies	North American region	N/A
Tabaeeian 2022	Healthcare professionals in general	N/A	Quantitative, qualitative, and mixed qualitative-quantitative studies	1. Telehealth, telemedicine, telemonitoring, and remote monitoring technologies	European, North American regions, Latin American and Australia	N/A
Tegegne 2023	Health professionals in general	2439	Quantitative and qualitative studies	1. Electronic medical records and clinical information systems	African region	N/A
Thomas Craig 2021	Physicians	9791	Quantitative and qualitative studies	1. CDSS 2. Electronic medical records and clinical information systems	European and North American regions	Burnout
Tickner 2023	Healthcare social workers	2599	Qualitative and quantitative studies	m-Health technologies 2. Telehealth, telemedicine, telemonitoring, and remote monitoring technologies	European and North American regions, and Australia	N/A
Torres-Castaño 2023	Healthcare professionals in general	N/A	Quantitative, qualitative, and mixed qualitative-quantitative studies	1. Telehealth, telemedicine, telemonitoring, and remote monitoring technologies	African, European, and North American regions, and Australia	Neurology
Vejdani 2022	Healthcare professionals in general	N/A	Quantitative, qualitative, and mixed qualitative-quantitative studies	1. Electronic medical records and clinical information systems	Asian, European, and North American regions	N/A
Verma 2022	Patients and Physicians	N/A	Quantitative, qualitative studies	1. Telehealth, telemedicine, telemonitoring and remote monitoring technologies	Asia, Europe, North American and Australia	N/A
von Wedel 2020	Physicians	N/A	Quantitative, qualitative, and mixed qualitative-quantitative studies	1. Electronic medical records and clinical information systems 2. CDSS 3. Advanced and business analytics 4. Telehealth, telemedicine, telemonitoring, and remote monitoring technologies	Asian, European, and Latin and North American regions, and Australia	N/A
Walle 2023	Healthcare professionals in general	1786	Quantitative studies	1. Electronic medical records and clinical information systems	African region	N/A
Walsh 2021	Ophthalmologists	N/A	N/A	1. Telehealth, Telemedicine, telemonitoring and remote monitoring technologies	European, and North American regions, and Australia and New Zealand	
Wisner 2019	Nurses and physicians	N/A	Quantitative, qualitative, and mixed qualitative-quantitative studies	1. Electronic medical records and clinical information systems	European and North American regions, and Australia	N/A
Xyrichis 2021	Healthcare professionals in general	268	Quantitative and qualitative studies	1. Telehealth, telemedicine, telemonitoring, and remote monitoring technologies	North American region	N/A
Young 2011	ICU staff	> 1325	Quantitative, qualitative, and mixed qualitative-quantitative studies	1. Telehealth, telemedicine, telemonitoring, and remote monitoring technologies	N/A	N/A
Zakerabasali 2021	Healthcare professionals in general	N/A	Qualitative and quantitative studies	1. m-Health technologies	African, European, and North American regions, and Australia and New Zealand	N/A
Zhang J 2023	Surgical trainees or qualified surgeons of any surgical specialty	N/A	Quantitative studies	1. Telehealth, telemedicine, telemonitoring, and remote monitoring technologies	N/A	Surgical field
Zhang Z 2023	Healthcare professionals in general	N/A	N/A	1. Telehealth, telemedicine, telemonitoring, and remote monitoring technologies 2. e-Health technologies	N/A	N/A

*CDSS* Computerized Decision Support Systems, *HIV* Human Immunodeficiency Virus, *ICD-10* International Classification of Diseases, 10th version, *ICU* Intensive Care Unit, *m-Health* mobile health technologies, *N/A* Not applicable or not available.

Few studies (*n* = 20; 18.5%) initially targeted evaluating the creation, implementation, long-lasting use, and self-reported barriers and facilitators to using digital health technologies by healthcare professionals^25,27,29,43,45,51,66,68,70,72–74,82,86,93,96,98,101,107,120^. Thus, the remaining reviews were cautiously evaluated in order to identify a report of any barrier or facilitator to using digital health technologies by healthcare workers. Included reviews were heterogeneous in terms of the digital health technologies being assessed (e.g., alert systems, clinical reminders applications, computerized clinical decision support systems, electronic documentation systems, mobile health applications, social media platforms, and telemedicine tools) and enrolling different healthcare professionals (e.g., general practitioners and specialists, nurses, pharmacists, community healthcare workers) at several levels of care (primary, secondary, and tertiary health facilities).

Most reviews (*n* = *63*; 58.3%) were executed in North America, Europe (*n* = *61*; 56.4%), and Asia (*n* = *50*; 46.2%). Thirty-three reviews suggested barriers and facilitators in the African territory (30.5%), while 28 reported data from Latin American and Caribbean regions (25.9%). Our study involved reviews from low- (e.g., Kenya, Rwanda, Uganda, and Ghana), middle- (e.g., Brazil, China, Russia, South Africa, and India), and high-income countries (e.g., Japan, the Czech Republic, United States of America, and Australia).

**Fig. 2:**
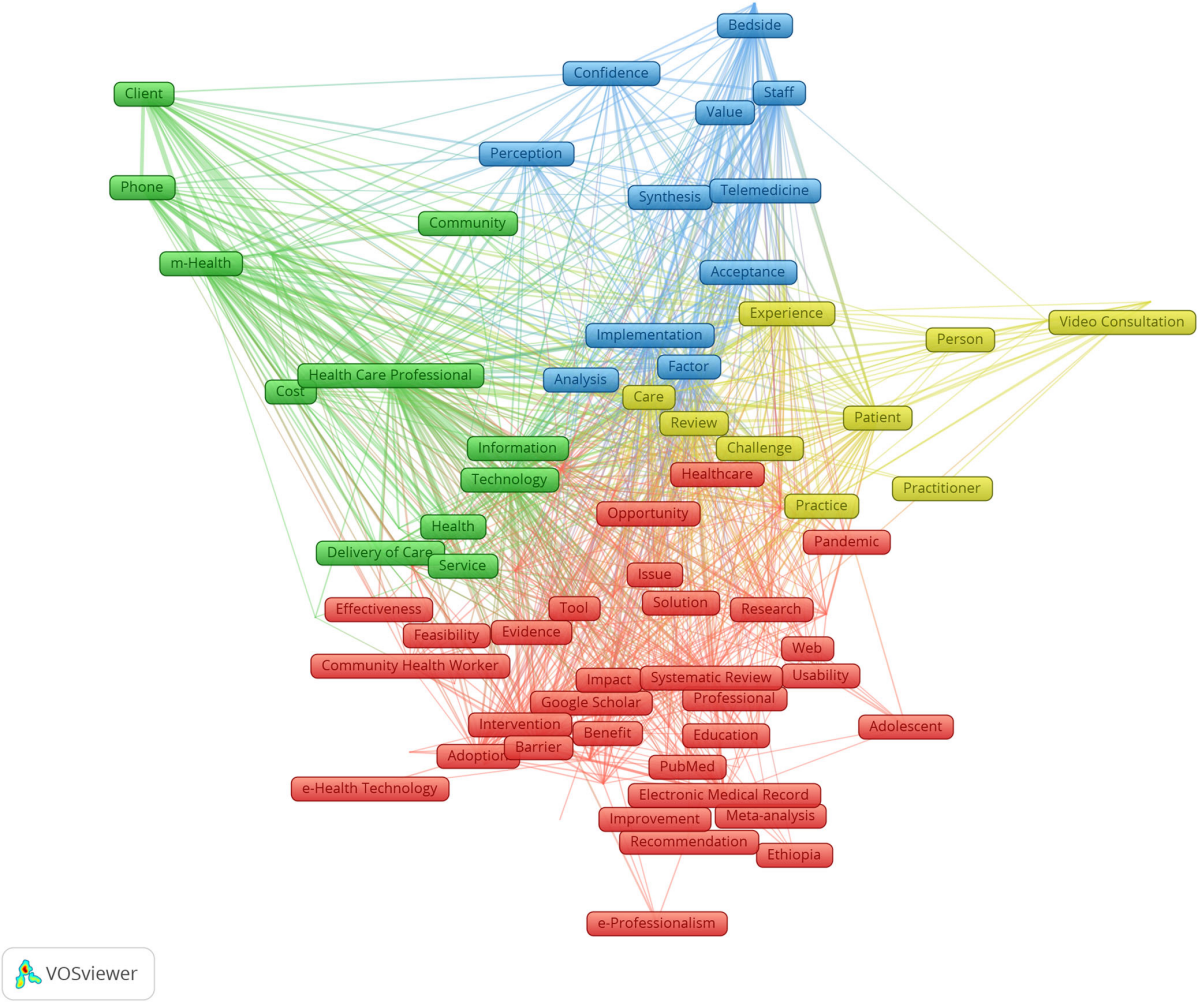
Overview of the network map of the most frequently identified terms among included studies. Please note that in the network visualization, items are represented by their label and by default also by a rectangles. The size of the label and the circle of an item is determined by the weight of the item. The higher the weight of an item, the larger the label and the circle of the item. The color of an item is determined by the cluster to which the item belongs.

According to our bibliometric analysis, our data were classified into five clusters based on identifier clustering assessment, and recorded keywords by co-occurrence frequency are shown in Table 3 and Fig. 2. The ten most common identifiers were “healthcare professionals,” “technology,” “review,” “barrier,” “care,” “systematic review,” “factor,” “patient,” and “implementation”.

**Table 3:** Top author-provided identifiers among included reviews.

Label	Cluster	Weight links	Weight as total link strength	Weight as occurrences
Acceptance	3	90	3216	48
Adolescent	1	20	516	12
Adoption	1	95	3044	88
Advantage	3	90	2076	24
Analysis	3	94	2448	43
Attitude	1	89	1086	22
Barrier	1	99	4539	125
Bedside	3	53	2785	15
Benefit	1	98	2203	48
Care	4	99	7425	125
Clinical Decision Support Systems	1	34	312	10
Challenge	4	100	3608	68
CINAHL	1	98	1507	29
Client	2	50	4947	25
Cochrane Library	1	81	422	11
Communication	1	96	2266	47
Community	2	84	1566	15
Community Health Worker	1	50	1296	36
Concern	3	91	1762	29
Confidence	3	83	2745	19
Cost	2	93	2524	28
COVID-19	1	89	1685	46
Data Collection	2	92	1555	22
Electronic Database	1	98	1684	44
Delivery of Care	2	93	1923	35
Depression	4	32	759	12
e-Health Technology	1	31	345	10
e-Professionalism	1	26	520	10
Education	1	88	1241	36
Effectiveness	1	91	924	19
Efficiency	1	83	542	15
Electronic Medical Record	1	89	2787	78
Embase	1	100	1618	33
Ethiopia	1	26	342	10
Evidence	1	100	2745	61
Experience	4	95	5895	71
Facilitator	1	90	1574	40
Factor	3	97	7144	112
Feasibility	1	76	489	15
Google Scholar	1	87	519	15
Health	2	96	2766	52
Health Care Professional	2	102	14681	176
Health Information Technology	1	68	669	23
Health Professional	1	84	1394	27
Healthcare	1	96	2149	45
Healthcare Professional	1	87	896	25
Healthcare Service	2	89	2888	26
Hospital Staff	3	49	2544	12
Impact	1	96	2566	66
Implementation	3	99	5893	95
Improvement	1	90	658	18
Information	2	100	3655	54
Integration	1	83	689	17
Intervention	1	89	3018	85
Issue	1	100	2654	49
Knowledge	1	101	2358	43
Literature Search	1	87	778	16
LMICs	2	61	609	16
Clinical Management	1	96	1486	31
MEDLINE	1	99	1914	41
Meta-analysis	1	85	862	24
m-Health	2	84	7650	76
Nurse	1	84	1350	46
Nursing	1	71	360	12
Opportunity	1	95	1470	26
Overview	1	74	510	15
Pandemic	1	90	1335	31
Patient	4	97	5055	97
Patient Care	1	82	592	20
Perception	3	88	3076	27
Person	4	85	1329	19
Phone	2	61	3817	24
Physician	1	94	2097	60
Practice	4	98	2898	66
Practitioner	4	77	664	16
PRISMA	1	87	566	16
Professional	1	85	962	25
PsycINFO	1	80	476	12
PubMed	1	98	1487	40
Recommendation	1	83	595	14
Research	1	100	2902	60
Review	4	100	8315	143
Science Direct	1	92	1110	25
Scoping Review	1	94	2050	60
Scopus	1	96	1142	31
Service	2	94	3020	62
Solution	1	79	630	17
Staff	3	87	5860	42
Strategy	1	94	1782	34
Synthesis	3	92	2154	21
Systematic Literature Review	1	75	596	17
Systematic Review	1	101	4086	116
Technology	2	99	7989	157
Telehealth	1	77	1237	31
Telemedicine	3	86	3127	34
Tool	1	97	2712	69
Training	1	99	3032	50
Treatment	4	82	860	15
Usability	1	77	798	18
Value	3	71	1494	14
Video Consultation	4	27	1202	22
Web	1	95	1320	30
Workflow	2	86	1055	18

Taking into account the 37 (34.2%) records providing data regarding the number of healthcare professionals considered in primary studies, sample sizes ranged from 22 to 106,876 (totaling approximately 345,000 healthcare workers), with a mean of 3,197 (SD 12,364), and a median of 1,545 (IQR 258 to 9,016). Most studies did not precisely consider one medical specialty, disease, or condition. However, some reviews focused on diseases of the respiratory system (e.g., tuberculosis, asthma, and chronic pulmonary obstructive disease)^19,22,31,32,46,93,101,123^, pregnancy, childbirth, or puerperium (e.g., maternal health, postpartum hemorrhage, and reproductive health)^19,22,23,26,31,35,46,56,61,77,94^, certain infectious or parasitic diseases (e.g., malaria, human immunodeficiency virus infection, and influenza)^19,22,23,28,31,46,50,61^, endocrine, nutritional, or metabolic diseases (e.g., diabetes mellitus)^57,64,76,93,123^, mental and behavioral disorders (e.g., post-traumatic disorder syndrome, stress, depression, and burnout)^23,41,44,64,70,76,94,125^, neoplasms^50,67,85,123^, diseases of the circulatory system (e.g., hypertension)^19,25,48,50,57,67,93,123^, diseases of the blood or blood-forming organs (e.g., anemia)^22^, and diseases or disorders of orofacial complex (e.g., oral lesions)^28,42^. Identified reviews mostly included quantitative (randomized and non-randomized trials, surveys, economic analysis, structured questionnaires, and experimental studies), qualitative (e.g., non-structured interviews, literature reviews, focus groups, observation, and cultural reports), and mixed-method reviews (sequential exploratory and concurrent transformative studies). An additional description of included reviews is shown in Table 2.

### Barriers and facilitators identified in included reviews and potential recommendations

**Fig. 3:**
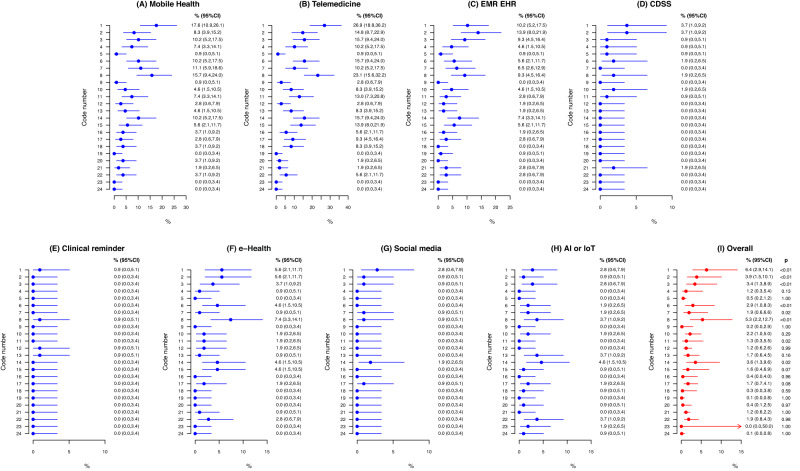
Relative frequency meta-analysis of most reported barriers for the use of digital health technologies by healthcare professionals. Frequencies (expressed as % and their confidence interval) are distributed among each categorized barriers as well as by healthcare technology modality.

**Fig. 4:**
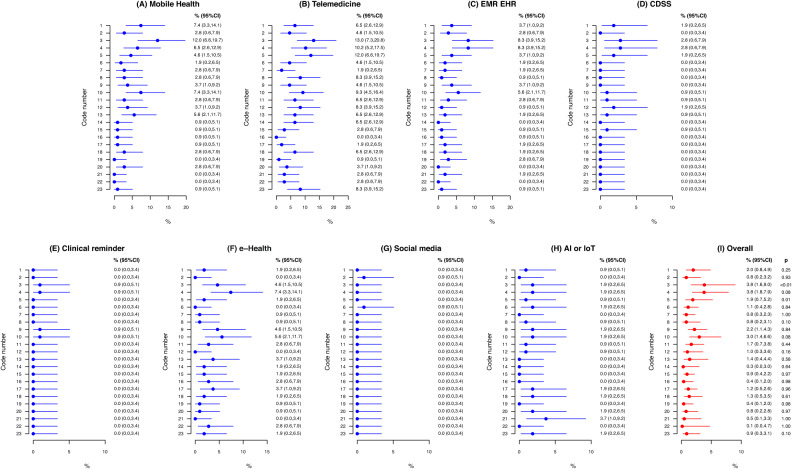
Relative frequency meta-analysis of most reported facilitators for the use of digital health technologies by healthcare professionals. Frequencies (expressed as % and their confidence interval) are distributed among each categorized facilitators as well as by healthcare technology modality.

The final domains created based on the thematic analysis can be accessed in Figs. 3, 4, and the summary of findings of the top seven barriers and facilitators can be accessed in Table 4. Our linguistic and semantic-based analysis stratified the data into 21 barriers and 19 recommendations. Predominant barriers were associated with infrastructure and technical (RFO of 6.4% [95% CI 2.9–14.1]), personal and psychological barriers (RFO of 5.3% [95% CI 2.2–12.7]), time and workload-related (RFO of 3.9% [95% CI 1.5–10.1]), training and educational (RFO of 3.4% [95% CI 1.3–8.9]), and legal- and ethical-related factors (RFO of 3.6% [95% CI 1.3–9.6]). Most predominant enablers related to the offer of training and educational activities (RFO of 3.8% [95% CI 1.6–9.0]), healthcare provider perception of digital health technologies usefulness and willingness to use (RFO of 3.8 % [95% CI 1.8–7.9]), the existence of government and multisector incentives (RFO of 3.0% [95% CI 1.4–6.6]), adherence promotion campaigns (RFO of 2.2% [95% CI 1.1–4.3]), involvement of healthcare providers in the process of digital health technologies development and implementation (RFO of 2.0% [95% CI 0.8–4.9]), and intuitive navigation in healthcare technology systems (RFO of 1.9% [95% CI 0.7–5.2]).

**Table 4:** Summary of qualitative findings.

Summary of review findings	RFO expressed as % (95% CI)	GRADE-CERQual components				
		Methodological Limitations^a^	Coherence^b^	Adequacy^c^	Relevance^d^	Overall assessment^e^
*Top 7 identified barriers*						
1. Healthcare professionals perceived that infrastructure and technical barriers were significantly crucial to using DHTs	6.4 % (95% CI 2.9–14.1)	Moderate concerns	No or very minor concerns	No or very minor concerns	No or very minor concerns	High confidence
2. Healthcare professionals perceived that psychological and personal issues directly affect the utilization of DHTs	5.3% (95% CI 2.2–12.7)	Moderate concerns	No or very minor concerns	No or very minor concerns	No or very minor concerns	High confidence
3. Fear of increased working hours and workload hinder the adoption and broad use of DHTs	3.9% (95% CI 1.5–10.1)	Moderate concerns	No or very minor concerns	No or very minor concerns	No or very minor concerns	High confidence
4. Healthcare professionals are aware and alert to legal and ethical features of using DHTs, factors that interfere with the success rate of any DHT	3.6% (95% CI 1.3–9.6)	Moderate concerns	No or very minor concerns	No or very minor concerns	No or very minor concerns	High confidence
5. Lack of training and educational programs causes a negative experience for healthcare professionals using DHTs, decreasing their use	3.4% (95% CI 1.3–8.9)	Moderate concerns	No or very minor concerns	No or very minor concerns	No or very minor concerns	High confidence
6. The structure of the healthcare system and lack of financial support limit the use of DHTs	2.9% (95% CI 1.0–8.3)	Moderate concerns	No or very minor concerns	No or very minor concerns	No or very minor concerns	High confidence
7. Interoperability and data incompatibility are conflicting elements in using DHTs	2.2% (95% CI 1.0–5.0)	Moderate concerns	No or very minor concerns	No or very minor concerns	No or very minor concerns	High confidence
*Top 7 identified facilitators*						
1. Offering training and educational activities increase the positive experience and facilitate the adoption of DHTs by healthcare providers	3.8% (95% CI 1.6–9.0)	Moderate concerns	No or very minor concerns	No or very minor concerns	No or very minor concerns	High confidence
2. Those healthcare professionals who perceived the full usefulness of DHTs and were willing and opened to the new technology are more likely to use them in a long-term period	3.8% (95% CI 1.8–7.9)	Moderate concerns	No or very minor concerns	No or very minor concerns	No or very minor concerns	High confidence
3. Government and multisector incentives increase the use of DHTs by healthcare professionals	3.0% (95% CI 1.4–6.6)	Moderate concerns	No or very minor concerns	No or very minor concerns	No or very minor concerns	High confidence
4. Adherence promotion campaigns facilitate and increase the use of DHTs by healthcare providers	2.2% (95% CI 1.1–4.3)	Moderate concerns	No or very minor concerns	No or very minor concerns	No or very minor concerns	High confidence
5. Involvement of healthcare professionals in the process of development and implementation of DHTs facilitates their experience with the technology and increases their acceptance	2.0% (95% CI 0.8–4.9)	Moderate concerns	No or very minor concerns	No or very minor concerns	No or very minor concerns	High confidence
6. Easy-to-use and intuitive navigation systems facilitate the use of DHTs by healthcare providers	1.9% (95% CI 0.7–5.2)	Moderate concerns	No or very minor concerns	No or very minor concerns	No or very minor concerns	High confidence
7. The existence of solid leadership and local champion facilitate the creation, implementation, and long-term adoption of DHTs by healthcare professionalsFeeling of reliability in utilized equipment and technologies improve the implementation and the adoption of DHTs by healthcare providers	1.7% (95% CI 0.7–3.8)	Moderate concerns	No or very minor concerns	No or very minor concerns	No or very minor concerns	High confidence

*CI* Confidence Interval, *DHTs* Digital Health Technologies, *RFO* Relative Frequency of Occurrence.

^a^We downgraded one level of confidence in the evidence based on the methodological quality of included systematic reviews and not based on the methodological limitations of primarily included studies. The rationale is that the AMSTAR-2 tool has seven strict critical domains, which, if occurred at least once, decreases overall confidence by two levels. Nevertheless, since several experts have already suggested that the reporting of many items in the PRISMA statement is suboptimal, we believe that this lack of reporting or evaluation might be associated with a “mass effect”, where researchers simply follow an inadequate pattern. Therefore, we decreased one level in the certainty of evidence instead of two levels on reviews’ methodological limitations.

^b^Coherence was rated as no or very minor concerns because the reviews’ findings appropriately described the data’s complexity, variation, and interconnectedness. Therefore, the available qualitative evidence provided no signs of contradictory, ambiguous, or incomplete data and competing theories or theoretical elements.

^c^We found the obtained data rich enough considering the complex and vast amount of data, the number of studies included, and their associated number of participants.

^d^Based on the review questions expressed in each included review, we judged the body of data from these reviews to be fully integrated with each research question.

^e^Although most of our included reviews were classified as “very low methodological quality” using the AMSTAR 2 tool, we believe that the reported data is significant enough not to decrease the confidence level primarily based on the methodological quality. We analyzed a group of phenomena that could hinder or enable the use of DHTs by healthcare providers, and we did find any signs of unbalanced or one-sided. Data underlying the reviews’ findings were sufficiently rich in terms of the number of studies and number of healthcare professionals.

As represented in Figs. 3, 4, several semantic clusters were described throughout included reviews. Herein, we outline and exemplify the five most common barriers and facilitators to the design, implementation, longitudinal maintenance, and evaluation of digital health technologies by healthcare professionals. The remaining barriers and facilitators are explained in detail in Supplementary Information 2 (pp 8). Infrastructure and technical barriers were the most frequently described barriers among included reviews, relating to issues with a limited or insufficient network, lack of existing technologies, lack of devices, compatibility with daily workflow, connectivity speed, healthcare capacity of technology integration, interconnectedness, absence of standardized/harmonized systems at different facilities, limited access to electricity, and requirement of a functional database system or large disk space. Notably, technical issues seem to be the worst in rural and countryside regions. Firstly, counteracting connectivity-related barriers involves ensuring availability (especially in rural areas) and affordability, guaranteeing high-speed fiber connectivity, and increasing the number of reliable local networks. In addition, we found reviews suggesting that to overcome infrastructure and technical barriers, the involvement of healthcare professionals in developing and implementing any health technology tools is fundamental, enhancing their capacity to manage such applications and increase their independence from co-workers and support centers. Remarkably, all reviews stated that user engagement and collaboration with system developers or associated stakeholders is crucial in all design and development stages, deployment, and continued utilization, as created applications are fit for purpose, based on understanding and addressing healthcare providers’ needs and expectations.

Personal and psychological barriers involved complex thematic components, including the healthcare professionals’ resistance to change, difficulty understanding the technology, perception of less human interaction, technophobia, ages, education levels, professional experience, low literacy, poor writing skills, linguistic features, adherence behavior, and fear of using particular health technology. Moreover, unwillingness, low expectations, skepticism from healthcare providers, and low motivation for compliance were also associated with personal barriers. For counterbalancing these barriers, healthcare professionals’ perception of usefulness and willingness was a highly cited facilitator, characterized by the degree to which the employees believe that using specific digital health technologies would enhance their performance and the proportion of participants intending to utilize that technology. Furthermore, personal and psychological barriers could be addressed by using and adopting training programs and educational activities appropriately tailored to healthcare professionals’ needs and coverage of deficient abilities. High-quality, real-time technical support and coaching also appeared as a component that increased healthcare providers’ efficiency, decreased implementation fear, and potentially could reduce internal conflicts during system adoption. Importantly, training programs may be developed with the ongoing involvement of the intended community to understand their needs and knowledge gaps. Moreover, evidence shows that user-friendly design, intuitive system navigation, and easy-to-use interfaces are critical to improving overall product performance and facilitating data collection and input, data processing, and further analysis.

Some reviews suggested that the limiting factors for the broad use of digital health technologies are associated with healthcare workers’ concerns about increased workload and altered workflow, which could hinder the sustainability of the digital health technologies. Additionally, these newly implemented technologies would require additional purchase time and increased set-up, implementation, training, access, adaptation, and establishment stages. In addition, healthcare professionals commonly stressed that digital health technologies would impact the quality of delivered care, as recently trained professionals would need a longer time to convert acquired data into the implemented system. However, although time might be required to acquire the right skills and operating competencies, with adequate training, continuous technical support, and peer-to-peer collaboration, threats associated with increased time to complete a specific task are significantly reduced. Useful written guidelines, instructions, and handouts appear to be important facilitators that could be easily implemented^73^. Likewise, incentives from government agencies and multisectoral organizations were shown to significantly improve digital health technologies’ effectiveness and chances of success in large-scale healthcare systems. Therefore, this conceptual perspective should be shown to healthcare providers, as increased effectiveness is directly related to the appropriate use of time and less wasteful processes.

Fourth, legal- and ethical-related barriers were shown to be a relevant factor for healthcare providers, as privacy and security concerns, national legislation, jurisdiction, and the existence of unclear legal liability regarding response protocols would directly affect healthcare professionals. Possible interventions for these barriers are associated with the development of safer data storage systems, the establishment of requirements on safety and security in cooperation with healthcare professionals and patients, or the creation of an international legal framework and legislative norm, which would clarify security regulation policies that could help ensure patients’ privacy and confidentiality, as well as define healthcare professionals’ liabilities.

Lastly, deficient or inexistent training and educational activities were evidenced to significantly impact the success and efficiency of digital health technologies in the healthcare environment . Some reviews highlighted that without training, healthcare providers tend to feel low self-efficacy when utilizing any digital health technologies, resulting in negative attitudes toward these technologies. In addition, as evidenced by healthcare workers, prior technology introduction, vendor training, in-depth seminars, workshops, or correlated training activities are unusual, and regular quality process assessment following implementation to ensure efficiency are also rare. Interestingly, reviews not only highlighted that training was fundamental to the success of using digital health technologies but also suggested that training per se would also be delivered through certain digital health technologies, such as mobile technologies and computers. Thus, the training offer positively affects healthcare professionals’ experience with digital health technologies, especially when monetary incentives are added to this variable, given the time invested in obtaining the proper abilities to operate any digital health technologies.

Using the AMSTAR 2 methodological quality assessment tool, most reviews had a very critically low overall methodological quality, as shown in Table 5. Nine-nine reviews were classified as very low quality, six as low quality, and only three were rated to have a high methodological quality. Two top-ranked reporting inadequacies related to the lack of evaluating the presence and likely impact of publication bias (95.2%), and the disregard of the risk of bias when interpreting the results of the review (95.2%). Where judgment was lost, this generally associated with the lack of prior protocol (50.9%), absence of justification for excluding individual studies (88.8%), lack of risk of bias assessment from individual studies being included in the review (63.8%).

**Table 5:** Quality assessment rating of systematic reviews included in the digital health solutions applied to healthcare workers environment overview.

Study ID	1	2	3	4	5	6	7	8	9	10	11	12	13	14	15	16	Overall Quality
Addotey-Delove 2023	Y	N	N	PY	Y	Y	N	N	N	N	NM	NM	N	N	NM	Y	Critically Low
Adepoju 2017	Y	N	Y	PY	Y	Y	N	PY	N	N	NA	NA	N	Y	NA	Y	Critically Low
Agarwal 2015	Y	N	Y	N	N	N	N	N	N	N	NA	NA	N	N	NA	N	Critically Low
Alkhaldi 2023	Y	N	Y	N	Y	Y	N	Y	Y	N	NM	NM	Y	Y	NM	Y	Critically Low
Al-Rawashdeh 2022	Y	Y	Y	Y	Y	Y	N	PY	Y	Y	NM	NM	Y	Y	NM	Y	Low
Amoakoh-Coleman 2016	Y	PY	Y	N	Y	Y	N	PY	PY	Y	NA	NA	Y	Y	NA	Y	Critically Low
Arsad 2023	Y	N	Y	N	N	N	N	Y	Y	N	NM	NM	N	N	NM	Y	Critically Low
Aslani 2022	Y	N	Y	N	N	Y	N	Y	N	N	NM	NM	N	N	NM	Y	Critically Low
Avoka 2022	Y	Y	Y	Y	Y	Y	N	Y	Y	Y	NM	NM	Y	Y	NM	Y	Low
Baluszek 2022	Y	Y	Y	Y	Y	Y	Y	Y	Y	Y	NM	NM	Y	Y	NM	Y	High
Bervell 2019	Y	N	N	Y	Y	N	N	PY	PY	N	NA	NA	N	N	NA	N	Critically Low
Boonstra 2010	Y	N	Y	N	Y	N	N	Y	N	N	NA	NA	N	N	NA	Y	Critically Low
Bommeyer 2023	Y	Y	Y	PY	Y	Y	N	PY	N	N	NA	NA	N	N	NM	Y	Critically Low
Braun 2013	Y	N	Y	N	Y	Y	N	N	N	N	NA	NA	N	Y	NA	Y	Critically Low
Brewster 2014	Y	N	Y	N	Y	N	N	Y	Y	N	NA	NA	N	N	NA	Y	Critically Low
Brown 2020	Y	PY	Y	N	N	N	N	PY	Y	N	NA	NA	Y	N	NA	N	Critically Low
Calleja 2022	Y	N	Y	N	Y	Y	N	Y	N	N	NM	NM	N	N	NM	N	Critically Low
Cansdale 2022	Y	N	Y	Y	Y	Y	N	Y	N	N	NM	NM	N	N	NM	Y	Critically Low
Cartolovni 2022	Y	N	Y	Y	Y	N	N	Y	N	N	NM	NM	N	N	NM	Y	Critically Low
Celes 2018	Y	PY	Y	PY	Y	Y	N	N	N	N	NM	NM	N	N	NM	Y	Critically Low
Cen 2022	Y	Y	Y	PY	Y	Y	N	Y	N	N	NM	NM	N	Y	NM	Y	Critically Low
Chen 2022	Y	N	Y	PY	N	N	Y	Y	Y	N	NM	NM	N	N	NM	Y	Critically Low
Chan 2018	Y	N	Y	N	Y	N	Y	Y	N	N	NA	NA	N	Y	NA	Y	Critically Low
Christensen 2020	Y	N	Y	PY	Y	N	N	PY	Y	N	NA	NA	Y	N	NA	Y	Critically Low
Da Costa 2020	Y	PY	Y	N	Y	Y	N	PY	N	N	NA	NA	N	N	NA	Y	Critically Low
Davis 2014	Y	N	Y	N	Y	N	N	PY	N	N	NA	NA	N	N	NA	Y	Critically Low
de Grood 2016	Y	N	Y	PY	Y	Y	N	Y	N	N	NA	NA	N	N	NA	Y	Critically Low
Drissi 2021	Y	N	N	N	N	N	N	PY	N	N	NA	NA	N	Y	NA	Y	Critically Low
Dutta 2020	Y	N	Y	N	N	N	N	N	N	N	NA	NA	N	N	NA	Y	Critically Low
Early 2019	Y	N	Y	N	Y	Y	N	N	N	N	NA	NA	N	Y	NA	N	Critically Low
Ebneter 2022	Y	N	Y	N	Y	Y	N	PY	N	N	NM	NM	N	Y	NM	Y	Critically Low
Emmett 2022	Y	PY	Y	N	Y	Y	N	PY	PY	N	NM	NM	Y	N	NM	Y	Critically Low
Ferdousi 2021	Y	Y	Y	N	Y	N	N	N	Y	N	Y	Y	Y	Y	Y	Y	Critically Low
Fletcher 2023	Y	N	Y	PY	Y	Y	N	Y	N	N	NM	NM	N	N	NM	Y	Critically Low
Ftouni 2022	Y	Y	Y	N	Y	Y	N	N	N	N	NM	NM	N	N	NM	Y	Critically Low
Gagnon 2012	Y	Y	Y	N	Y	Y	N	PY	Y	N	NA	NA	Y	Y	NA	N	Critically Low
Gagnon 2016	Y	N	Y	N	N	Y	Y	Y	N	N	NA	NA	N	N	NA	Y	Critically Low
Garavand 2022	Y	N	N	Y	N	Y	N	PY	Y	Y	NM	NM	N	N	NM	Y	Critically Low
Garvey 2021	Y	N	N	PY	N	N	N	Y	Y	Y	NM	NM	Y	Y	NM	Y	Critically Low
Ghimire 2023	Y	Y	Y	Y	Y	Y	N	Y	Y	Y	NM	NM	Y	Y	NM	Y	Low
Gonçalves R 2023	Y	PY	Y	Y	Y	Y	Y	Y	Y	N	NM	NM	Y	N	NM	Y	Critically Low
Grant 2022	Y	Y	Y	Y	Y	Y	N	Y	Y	N	NM	NM	Y	N	NM	Y	Critically Low
Hagstram 2022	Y	Y	Y	PY	Y	Y	N	N	N	N	NM	NM	N	N	NM	Y	Critically Low
Huang 2023	Y	PY	Y	N	Y	Y	N	PY	Y	N	NM	NM	Y	N	NM	Y	Critically Low
Ionescu 2022	Y	N	Y	N	Y	N	N	PY	N	N	NM	NM	N	N	NM	Y	Critically Low
Isidori 2022	Y	N	Y	N	N	N	N	PY	N	N	NM	NM	N	N	NM	Y	Critically Low
Ismatullaev 2022	Y	N	Y	N	Y	Y	N	N	N	N	NM	NM	N	N	NM	N	Critically Low
Jacob 2020	Y	PY	Y	PY	Y	N	N	N	Y	N	NM	NM	N	N	NM	Y	Critically Low
Jimenez 2022	Y	Y	Y	N	Y	Y	N	N	N	N	NM	NM	N	N	NM	Y	Critically Low
Jimma 2022	Y	Y	Y	PY	Y	Y	Y	PY	N	N	NM	NM	N	N	NM	Y	Critically Low
Joo 2022	Y	N	Y	N	N	N	N	N	N	N	NM	NM	N	N	NM	Y	Critically Low
Jonasdottir 2022	Y	Y	Y	Y	Y	Y	N	PY	N	N	NM	NM	N	N	NM	Y	Critically Low
Jose 2023	Y	N	N	PY	Y	N	N	N	N	Y	NM	NM	Y	Y	NM	Y	Critically Low
K. Zhang 2022	Y	N	Y	N	N	N	N	N	N	N	NM	NM	N	N	NM	Y	Critically Low
Kane 2022	Y	N	Y	N	Y	N	N	N	N	N	NM	NM	N	N	NM	Y	Critically Low
Keyworth 2018	Y	PY	Y	PY	Y	N	N	PY	N	N	NM	NM	N	N	NM	Y	Critically Low
Koivunen 2018	Y	Y	N	Y	N	N	N	PY	Y	N	NM	NM	N	Y	NM	Y	Critically Low
Kolla 2021	Y	N	Y	PY	N	N	N	Y	N	N	NA	NA	N	N	NA	Y	Critically Low
Konnyu 2023	Y	Y	Y	PY	Y	Y	N	PY	N	N	NM	NM	N	N	NM	Y	Critically Low
Kruse 2022	Y	PY	N	Y	Y	Y	N	PY	Y	N	NM	NM	Y	N	NM	Y	Critically Low
Laar 2022	Y	Y	Y	Y	Y	Y	N	PY	Y	N	NM	NM	N	N	NM	Y	Critically Low
Lam 2022	Y	N	N	N	N	N	N	PY	N	Y	NM	NM	Y	Y	NM	Y	Critically Low
Lampickien 2022	Y	N	Y	N	N	N	N	N	N	N	NM	NM	N	N	NM	Y	Critically Low
Li 2013	Y	N	Y	N	N	N	N	Y	N	N	NA	NA	N	N	NA	Y	Critically Low
Li 2019	Y	PY	Y	Y	N	N	N	Y	Y	N	NA	NA	Y	N	NA	Y	Low
Lluch 2020	Y	N	Y	PY	N	N	N	N	N	N	NA	NA	N	N	NA	Y	Critically Low
Longhini 2022	Y	Y	Y	Y	Y	Y	N	Y	Y	N	NM	NM	N	N	NM	Y	Critically Low
Martin 2019	Y	Y	Y	Y	Y	Y	N	Y	Y	N	NA	NA	N	Y	NA	Y	Critically Low
Marvaso 2022	Y	PY	N	PY	N	N	N	PY	N	Y	NM	NM	N	N	NM	Y	Critically Low
Meunier 2023	Y	Y	Y	PY	Y	Y	N	PY	Y	Y	NM	NM	Y	Y	NM	Y	Low
Moore 2020	Y	N	Y	PY	Y	Y	N	Y	Y	N	NM	NM	Y	N	NM	Y	Critically Low
Muhiyaddin 2020	Y	N	N	PY	Y	Y	N	N	N	N	NA	NA	N	N	NA	N	Critically Low
Mulita 2022	Y	N	N	PY	N	N	N	N	N	Y	NM	NM	N	Y	NM	Y	Critically Low
Namasivayam 2022	Y	Y	Y	PY	Y	Y	N	Y	N	Y	NM	NM	N	Y	NM	Y	Critically Low
Nezamdoust 2022	Y	N	Y	PY	N	N	N	N	N	Y	NM	NM	N	Y	NM	Y	Critically Low
Nguyen 2021	Y	Y	Y	N	Y	Y	N	Y	N	N	NA	NA	N	Y	NA	Y	Critically Low
Niazkhani 2020	Y	N	Y	N	Y	Y	N	Y	Y	N	NA	NA	N	N	NA	Y	Critically Low
Nizeyimana 2022	Y	Y	Y	Y	Y	Y	N	N	N	N	NA	NA	N	N	NA	Y	Critically Low
O’Connor 2022	Y	N	Y	N	Y	N	N	N	N	N	NM	NM	N	N	NM	Y	Critically Low
Odendaal 2020	Y	Y	Y	Y	Y	Y	Y	Y	Y	N	NA	NA	Y	Y	NA	Y	High
Osman 2019	Y	Y	Y	N	Y	N	N	Y	Y	N	NA	NA	N	N	NA	Y	Critically Low
Papadopoulos 2018	Y	N	Y	N	Y	Y	N	PY	N	N	NM	NM	N	Y	NM	N	Critically Low
Police 2011	Y	N	N	Y	N	N	N	PY	N	N	NA	NA	N	N	NA	Y	Critically Low
Prakash 2022	Y	Y	Y	Y	Y	Y	N	N	N	N	NA	NA	N	N	NA	Y	Critically Low
Rahal 2021	Y	Y	Y	N	Y	Y	N	Y	Y	N	NA	NA	Y	N	NA	Y	Critically Low
Ramachandran 2023	Y	Y	Y	Y	Y	Y	N	Y	N	N	NA	NA	N	N	NA	Y	Critically Low
Ratshidi 2022	Y	Y	Y	PY	N	N	N	N	N	N	NA	NA	N	N	NA	Y	Critically Low
Rukavina 2021	Y	Y	Y	N	Y	Y	Y	Y	N	N	NA	NA	N	N	NA	Y	Critically Low
Saigi-Rubio 2022	Y	Y	Y	N	Y	Y	N	Y	Y	Y	NM	NM	Y	N	NM	Y	Critically Low
Sipanoun 2022	Y	Y	Y	Y	Y	Y	Y	Y	Y	N	NM	NM	Y	Y	NM	Y	Low
Sullivan 2022	Y	N	N	N	N	N	N	PY	N	N	NM	NM	N	N	NM	Y	Critically Low
Poissant 2005	Y	N	Y	Y	Y	N	N	Y	N	N	NA	NA	N	Y	NA	Y	Critically Low
Tabaeeian 2022	Y	N	Y	N	Y	Y	N	N	N	N	NM	NM	N	N	NM	N	Critically Low
Tegegne 2023	Y	PY	N	PY	N	Y	N	Y	N	Y	Y	N	N	Y	Y	Y	Critically Low
Thomas Craig 2021	Y	Y	Y	Y	Y	Y	N	Y	Y	N	NA	NA	N	Y	NA	Y	Critically Low
Tickner 2023	Y	N	Y	Y	N	N	N	Y	N	N	NM	NM	N	N	NM	Y	Critically Low
Torres-Castano 2023	Y	Y	Y	Y	Y	N	Y	Y	N	N	NM	NM	N	N	NM	Y	Critically Low
Vejdani 2022	Y	PY	Y	PY	Y	N	N	Y	N	N	NM	NM	N	Y	NM	Y	Critically Low
Verma 2022	Y	Y	Y	N	Y	N	N	Y	N	N	NM	NM	N	N	NM	Y	Critically Low
Von Wedel 2020	Y	PY	N	Y	Y	N	Y	Y	N	N	NA	NA	N	Y	NA	Y	Critically Low
Walle 2023	Y	PY	Y	PY	Y	N	N	Y	Y	Y	Y	Y	Y	Y	Y	Y	Critically Low
Walsh 2021	Y	N	Y	PY	N	N	N	Y	N	Y	NM	NM	N	Y	NM	Y	Critically Low
Wisner 2019	Y	PY	Y	Y	N	N	N	PY	Y	N	NA	NA	N	N	NA	N	Critically Low
Xyrichis 2021	Y	Y	Y	Y	Y	Y	Y	Y	Y	N	NA	NA	Y	Y	NA	Y	High
Young 2011	Y	N	N	Y	Y	N	N	Y	N	N	NA	NA	N	Y	NA	Y	Critically Low
Zakerabasali 2021	Y	N	Y	N	Y	Y	N	Y	N	N	NA	NA	N	N	NA	Y	Critically Low
Zhang J 2023	Y	N	N	N	Y	N	N	N	N	N	NM	NM	N	N	NM	Y	Critically Low
Zhang Z 2023	Y	N	Y	N	Y	Y	N	N	N	N	NM	NM	N	N	NM	Y	Critically Low

Judgement was performed by two reviews authors and based on the AMSTAR-2 approach.

Domain 1—Did the research questions and inclusion criteria for the review include the components of PICO?

Domain 2—Did the report of the review contain an explicit statement that the review methods were established prior to the conduct of the review and did the report justify any significant deviations from the protocol?

Domain 3—Did the review authors explain their selection of the study designs for inclusion in the review?

Domain 4—Did the review authors use a comprehensive literature search strategy?

Domain 5—Did the review authors perform study selection in duplicate?

Domain 6—Did the review authors perform data extraction in duplicate?

Domain 7—Did the review authors provide a list of excluded studies and justify the exclusions?

Domain 8—Did the review authors describe the included studies in adequate detail?

Domain 9—Did the review authors use a satisfactory technique for assessing the risk of bias (RoB) in individual studies that were included in the review?

Domain 10—Did the review authors report on the sources of funding for the studies included in the review?

Domain 11—If meta-analysis was performed did the review authors use appropriate methods for statistical combination of results?

Domain 12—If meta-analysis was performed, did the review authors assess the potential impact of RoB in individual studies on the results of the meta-analysis or other evidence synthesis?

Domain 13—Did the review authors account for RoB in individual studies when interpreting/ discussing the results of the review?

Domain 14—Did the review authors provide a satisfactory explanation for, and discussion of, any heterogeneity observed in the results of the review?

Domain 15—If they performed quantitative synthesis did the review authors carry out an adequate investigation of publication bias (small study bias) and discuss its likely impact on the results of the review?

Domain 16—Did the review authors report any potential sources of conflict of interest, including any funding they received for conducting the review.

**Fig. 5:**
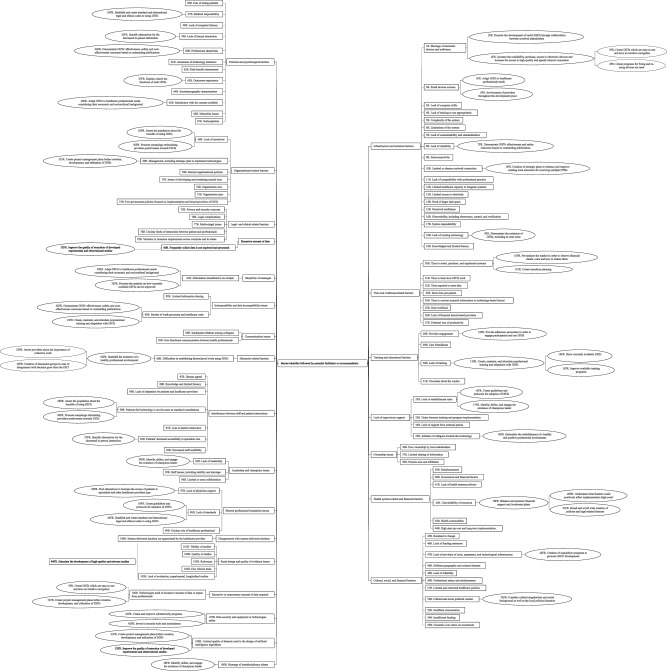
Conceptual map of reported barriers and potential facilitators and recommendations to overcome these barriers.

We mapped the aforementioned data and complementary results, as shown in Fig. 5 (also available for virtual access through the GitMind platform).^126^ As evidenced in supplementary information 3 (pp 9), we found several terms with similar semantic structures. Thus, we coded each barrier or facilitator and identified recommendations, suggesting the possibility of a complex and broad linguistic connection and relationship amongst codes. These thematic relationships are not limited in our analysis and can be explored and exhausted.

## Discussion

To our knowledge, this is the first overview of systematic reviews to collate, cluster, and synthesize the quantitative, qualitative, and mixed methods body of literature associated with barriers and facilitators to and use of several digital health technologies by healthcare professionals at all levels of care. The decision for carrying out this valuable, but complex study, relies on the noticeable detachment of research data and investigation groups in the field of Medical Informatics, who usually inadvertently duplicate technical and financial resources given the existing gaps in the literature. Here we report 21 overarching barriers and 19 facilitators, mostly interconnected, containing a complex sequence of thematic describers and identifiers. Understanding and overcoming identified barriers to the fully integrated and coordinated use of DHTs by any class of healthcare providers and evaluating its facilitators could positively impact successful creation, implementation, adoption, training, and long-term services or product utilization.

The evidence suggests that healthcare providers and managers predominantly face infrastructure, technical-, training-, legal-, ethics-, time-, and workload-related barriers to using digital health technologies, regardless of the level of care or digital technology. In the second level of semantic occurrence, several restraining factors to the wide use of digital health technologies were combined and reported, including psychological and personal barriers, lack of supervisory support, ownership issues, and healthcare system-cultural-, social-, and financial-related limiting features. Nevertheless, we are aware that some of the classified items are interconnected, meaning that the prevalence of occurrence ranking should not be used as a priority guide for policymakers and health organizations when addressing these barriers. For instance, the highlighted barrier “81B” (regarding the simplicity of contents usually transferred in mobile applications or clinical alert systems) might be directly related (or potentially caused due to) to the technical limitations per se (considering devices screen’s reduced size (“2B”), the complexity of the systems themselves and the information they carry (“5B”), or even because the lack of standardization and customizability of such systems and technologies (“7B”). Therefore, the creation of artificial intelligence-based mind mapping representing these interconnections is of utmost relevance^126^.

Creating and applying digital health technologies to healthcare environments must be driven by a regime of comprehensive assumptions instead of empirical models and processes. Our results corroborate with published systematic reviews that have already evidenced patient-reported barriers and facilitators to utilizing digital health solutions for self-care^127–129^. For instance, self-management of low-back pain using mobile health applications was mainly challenging due to information technology, usability-accessibility, quality-quantity of content, tailoring-personalization, and motivation-support barriers^127^. In contrast, flexibly structured and intuitive navigation, trustworthy content and sources, content accounting for individual needs and priorities, and the opportunity to influence the application design appeared as relevant facilitators affecting the uptake and utilization of digital health interventions for self-management of lower back pain^127^. Likewise, Powell and colleagues suggested that a lack of awareness, self-motivation, training, privacy, and security concerns are the most common patient-derived barriers to using electronic portals^128^. Emphasized facilitators correlated with use engagement by a leader (i.e., physician), free access and control over health information, and an adequate communication profile. Therefore, as the relationships between our identified barriers and facilitators and existing patient-related evidence highlight, the development of digital healthcare solutions should consider multiple factors, which can facilitate or deteriorate broad goals of high-quality use of information technology in the healthcare environment.

During protocol modeling, our research group discussed the possibility of including reviews that summarize evidence on barriers and facilitators involving students in health fields. The decision was not to include these reviews because these students are not yet legally considered professionals or critically necessary workforce, and they are not considered essential in healthcare settings^130,131^. However, one aspect found in these excluded reviews was revealed in our overview with significant frequent and relevant findings: the use of digital health technologies for training and educational purposes. Although distance education dates from 1728^132,133^, e-learning or virtual learning started during the early 1980s at the University of Toronto^134^ and has been developing ever since, particularly during the COVID-19 pandemic^135,136^. Currently, several high-income countries, such as New Zealand and the United States of America, have already integrated and implemented the Information and Communication Technology constructivist learning model in their national or statewide policies, ensuring that students have the chance to become digitally competent citizens^137,138^. These actions effectively decrease multiple barriers observed related to limited or no computer skills, restricted knowledge and technology literacy, and lack of reliability in technological tools. However, it has been suggested that numerous low- and middle-income countries still struggle with device acquisition, connectivity issues, tutors’ level of expertise and lack of motivation, absence of basic infrastructure, and the unwillingness of the government to implement such solutions^129^.

Foremost, we chose only six health solutions as systematic and feasible choices for comprehensive data processing. Nevertheless, we observed additional modalities of health solutions being implemented worldwide (e.g., laboratory and radiology automatic reporting systems, picture archiving and communication systems, cloud-based systems, and advanced and business analytics), and our synthesis may miss emerging or recent technologies^52,74,114^. For instance, studies have suggested that electronic laboratory reporting systems not only improve surveillance for notifiable conditions but can also be helpful in real-time laboratory testing in emergency departments and significantly improve organizational framework and efficiency^139,140^. Correspondingly, cloud-based computing systems have been increasingly applied in the healthcare system to ensure secure storage, handling, and processing of medical information^141^. Regardless of the digital health solution being implemented and utilized, healthcare workers and patients benefit from it. By improving real-time patient access to their results and providing better patient involvement with care, the incidence of unwanted tests or extra prescriptions decreases, and the overall quality of care is subsequently enhanced^142,143^.

We observed a limited number of reviews assessing the potential challenges and enablers for artificial intelligence models, machine learning algorithms, and platforms utilizing features such as augmented reality^40,54,63,70,78,85,94,99^. However, although the restricted number of studies assessing these subgroups in the field of digital technologies, core barriers and facilitators remained like other subgroups. Nevertheless, we highlight the need for further research with these technologies, as alternative barriers and facilitators would arise.

Due to the wide variety of digital health technologies currently being used in several medical specialties and levels of care, we had to restrict our report in different ways, limiting our certainty of evidence. Similarly, our series of analyses did not consider the existence of subgroup singularities by type of healthcare professional. As suggested in our map based on bibliometric data, only physicians, community health workers, and nurses appeared as recurrent keywords among all studies within the 42 systematic reviews eligible for inclusion. Therefore, studies analyzing impeding and enabling factors to the general use of digital health technologies in other healthcare providers (e.g., pharmacists, physiotherapists, physical educators, speech therapists, healthcare governmental agents, biologists, social services agents, healthcare managers, dentists, and psychologists) cause a “professional class bias” event that should be addressed in future studies. Likewise, factors like age, racial group, gender, country income index, or geographic location could affect a different subgroup (e.g., potential higher reporting of barriers of professionals practicing in low- or middle-income countries would focus more on technical and infrastructure features). Moreover, we neglected that digital health technologies utilized in the healthcare environment are usually concomitant and integrated. Thus, we may have considered the reported health solution independently instead of using a translational and adapted assignment methodology. Therefore, the provided RFO represented only the tendency of domain observance and reporting and not the identical picture of healthcare professionals’ reality. To conclude, we are aware that some highlighted barriers and facilitators could be assigned to a broader subtheme (e.g., lack of supervisory support in training and educational skills). However, during the overall execution, we observed that some terminologies and coding were commonly reported separately, so we decided to maintain them as individual elements to ensure the representativeness of the findings. Interestingly, the use of the AMSTAR 2 tool for evaluating the methodological quality of all included reviews should also be stated as a limitation, as the approach was primarily intended to systematic reviews of randomized controlled trials. Nevertheless, as most AMSTAR domains are on the elements that any review is structured (e.g., search strategy, protocol, extraction, combing studies, and publication bias), we believe that applying this methodology to our include reviews do not hinder the observed results. Likewise, although we Apart from these minor methodological limitations, the major strength of our study is the strict adhesion to international guidelines for reporting of systematic reviews (e.g., Preferred Reporting Items for Systematic Reviews and Meta-Analyses statement and the Cochrane Handbook of Systematic Reviews and Meta-Analyses) and the execution of the entire study with international and blinded collaboration. We acknowledge that more than one methodology for evaluating the certainty of the evidence in qualitative research exists. We applied the GRADE CERQual method to check the overall quality of evidence for the seven most-reported barriers and facilitators. Generally, the evidence quality is high, with all considered domains without major concerns but with methodological limitations. We judged this domain as a moderate concern based on the phenomena of interest, adequate data collection and extraction, and quality in reporting observed data. In addition, expert groups have been discussing.

Although digital health technologies and their numerous types of technologies positively affect the healthcare environment, barriers impacting the successful creation, adoption, implementation, and sustainability of digital interventions are commonly reported by healthcare workers. Notwithstanding, the identification and deployment of different enabling factors allow the utilization of digital technologies in a holistic and integrated way. This overview of reviews emphasizes remarkable limiting features that should be considered by all stakeholders and provides advice to overcome these issues, with the expectation of increasing professional satisfaction and, perhaps, the quality of delivered care.

## Methods

This overview of systematic and scoping review (herein referred to as “overview”) protocol was registered on PROSPERO (CRD42022304372, supplementary information 4, pp 10–20) and it was part of a broader study conducted by the Data and Digital Health Unit of the Division of Country Health Policies and Systems of the World Health Organization, Regional Office for Europe^3^. This initiative provides strategic direction, technical assistance, and tailored support to countries and policymakers to strengthen their capacity to generate timely, credible, reliable, and actionable health-related data. The scientific community is currently defining an explicit, systematic, and transparent methodology to create evidence- and agreement-based reporting guidelines for overviews of reviews^144^. Therefore, we used the Preferred Reporting Items for Systematic Reviews and Meta-Analysis reporting recommendations^145^, the Cochrane Handbook guidelines^146^, and reports published by Fusar-Poli et al.^147^ and Cornell et al.^148^ guiding the practice on how to effectively conduct an umbrella review. As our study relies upon secondary data, ethics approval was waived. It is worthwhile mentioning that although in our protocol we initially stated that a standard meta-analysis would not be carried out, we decided to mathematically evaluate the obtained results. The technique utilized for the word- and sentence-based assessment (particularly associated with discourse analysis) is a well-known summarizing strategy used in the field of Human Sciences and was systematically presented and implemented in our research team after the protocol preparation. Therefore, in consonance with the requirements of continuous scientific evolvement and improvement, we decided to apply this newly introduced technique. However, this deviation does not alter the core of this project.

### Data sources and searches

We searched five databases (Cochrane Database of Systematic Reviews, Embase^®^, Epistemonikos, MEDLINE^®^, and Scopus) and the PROSPERO protocol registration platform from inception to Jan 23, 2022, for systematic and scoping reviews evaluating barriers and facilitators to using digital health technologies by healthcare professionals worldwide. We also performed a manual search of reference lists of reviews shortlisted for full-text review and planned to contact the authors of included review to retrieve additional data.

An experienced information specialist and the expert team tailored search strategies to each database using Medical Subject Headings (MeSH) and free-text identifiers associated with the research topic^149–152^. The search included three main categories of key terms. Digital health technologies search identifiers included terms such as “telemedicine,” “telehealth,” “mobile health,” “mHealth,” “artificial intelligence,” “machine learning,” “social media,” “natural language processing,” and “computer decision support systems,” healthcare professional-related terms included “healthcare worker,” “healthcare provider,” and “healthcare support worker,” and systematic review filters used were “systematic review,” “meta-analysis,” and “scoping review.” Our terms are defined in recently published studies in the World Health Organization guidelines on digital health technologies for strengthening health systems, the World Assembly Resolution on Digital Health, and The Lancet Digital Health. In supplementary information 5 (pp 21-28), we present the detailed search strategy for the databases.

### Study selection

Eligibility was evaluated by two independent investigators who primarily screened titles and abstracts and subsequently reviewed the full texts using Covidence^®^ (Veritas Health Innovation, Melbourne, Australia)^153^. Systematic and scoping reviews deemed eligible must have used at least two databases for their assessment, should have described the search methods, and evidenced the use of a transparent methodology for study selection and data extraction. Moreover, these reviews were only included if a qualitative analysis of barriers and facilitators to using digital health technologies by healthcare providers was clearly noted. We did not place limits on targeted healthcare professionals, medical specialty, level of care, language, and publication date. However, in order to avoid bias and results inflation, those studies strictly prioritizing the assessment of digital technologies for students and education in the field of health sciences were excluded.

### Data extraction and quality assessment

Two independent researchers appraised the methodological quality of included systematic reviews using the AMSTAR-2 tool^154^. Following the initial evaluation, a third researcher cross-checked rated domains. The methodological quality of reviews was classified as “critically low,” “low,” “moderate,” and “high.” Our research team is aware that the AMSTAR 2 tool is not intended to generate an overall score of the review’s quality. Thus, we emphasize that we considered the appraisal methodology holistically, mostly related to the provision of an extensive evaluation of quality, particularly weaknesses associated with poor conduct of the review or word counting limitation endorsed by a determined journal.

Relevant data (first author identification, publication year, published journal, number of included databases, review objectives, primary study design, type of healthcare professional, type of digital technologies being analyzed, number of included primary studies, and barriers, facilitators, and recommendations for using digital health technologies) was extracted from included reviews by two independent researchers using Microsoft Excel (Microsoft Corporation, Redmond, USA)^155^. In the second stage, four independent volunteer collaborators reassessed extracted data to resolve inconsistencies.

### Data synthesis and analysis

We used VOSviewer to assess research hotspots associated with digital health technologies based on the principle of co-occurrence analysis^156^. The minimum number of co-occurrences was set as 3, normalization method as an association, random starts as 1, random seed as 0, resolution as 1, and we merged small clusters. We attempted to clean the network map as much as possible, as some keywords were not meaningful. Thus, we extracted data from the top 100 author-provided keywords and mapped them into a single keyword co-existing network. Representative and frequent terms are expressed as larger nodes, and the thickness of the link between two or more nodes represents the strength of the relationships between them.

Our findings were evaluated and collated using an adapted version of a thematic synthesis developed by Thomas and Harden^157^. The 21 domains prioritized in the Enhancing Transparency in Reporting the Synthesis of Qualitative Research (ENTREQ) statement were followed^158^. First, qualitative data of included reviews on the main barriers and facilitators identified were coded line-by-line using QSR’s NVivo software (QSR International, Burlington, USA)^159^. In addition, primary highlighted concepts were re-evaluated by four volunteer collaborators who double-checked selected data and evaluated extraction errors or missing information. If needed, they also created new in-text selections. Furthermore, we organized free selections into similar themes to combine the preliminary results into descriptive themes. Lastly, we developed analytical themes that summarized barriers and facilitators closely related to the original remarks reported in included reviews. The explanatory delineation of thematic barriers and facilitators was a dynamic, deductive, and intuitive process, as different review authors had their peculiarities in academic and text writing. The alignment of thematic barriers and facilitators was discussed by all authors, resulting in the development of recommendations. In the result section, we have identified only the five most frequent barriers and facilitators. Recommendations were also emphasized for these five features. However, a complete list of barriers, facilitators, and recommendations can be accessed in supplementary information 2 (2.1 and 2.2). Where homogenous barriers were recognized (e.g., lack of leadership and local champions), guidance to overcome these barriers were prepared by the group of specialists (e.g., identification of processes weaknesses, implementation of improved strategies, and adjustment of progress based on stakeholder feedback). Similarly, the recommendations also considered the identified facilitators. Systematic reviews with similar research questions were expected to be included in our umbrella review. Consequently, the likelihood of two or more reviews including the same primary study in their analysis was meaningful^160^. Therefore, we carefully extracted and evaluated all references mentioned in the results section of each included review to exclude overlapping studies.

After establishing analytical themes, the frequency of occurrence for each categorized barrier and facilitator was aggregated into a standard meta-analysis of proportions. Certainty of the evidence was based on the GRADE-Cer-Qual approach^161^. Nominally identified results are indicated as the relative frequency of occurrence (RFO) and 95% confidence interval (CI). Analysis was executed using R software (version 4.1.1), using the metaprop function package. This study is deemed exempt as it does not assess data or intervene in humans.

### Supplementary information

Supplementary Material

## Data Availability

The authors hereby declare that all pertinent data has already been displayed within the article. Additional data can be accessed upon request to Dr. Israel Júnior Borges do Nascimento (borgesi@who.int) or Dr. David Novillo-Ortiz (dnovillo@who.int).
