# Supplementary material for: Barriers and facilitators to utilizing digital health technologies by healthcare professionals
Source: NPJ Digit Med. 2023 Sep 18;6:161. doi: 10.1038/s41746-023-00899-4 (PMC10507089; doi:10.1038/s41746-023-00899-4)
Supplement: Supplementary file 1 — Supplementary Material [file 41746_2023_899_MOESM1_ESM.pdf]

Table of Contents

SUPPLEMENTARY INFORMATION 1– LIST OF EXCLUDED REVIEWS WITH JUSTIFICATION 2

SUPPLEMENTARY INFORMATION 2 – LIST OF BARRIERS AND FACILITATORS CATEGORIZED IN THE REVIEW 8

SUPPLEMENTARY INFORMATION 3. FIGURE LEGEND AND EXPLANATION OF POTENTIAL CONNECTIONS/RELATIONSHIPS OF IDENTIFIED BARRIERS 9

SUPPLEMENTARY INFORMATION 4 – PROTOCOL REGISTERED ON PROSPERO 10

SUPPLEMENTARY INFORMATION 5 – SEARCH STRATEGIES 21

Supplementary Information 1– List of excluded reviews with justification

	Title	Authors	Published Year	Journal	Exclusion reason
1	The use and impact of mHealth by community health workers in developing and least developed countries: a systematic review	Abreu et al	2021	Res. Biomed. Eng.	3
2	Barriers for adopting electronic health records (EHRs) by physicians	Ajami et al	2013	Acta Informatica Medica	4
3	The impact of artificial intelligence on radiography as a profession: A narrative review	Al-Naser et al	2022	Journal of medical imaging and radiation sciences	4
4	Telemedicine and its impact on rural health care in times of COVID-19: A systematic review	Albornoz-Chauca et al	2022	Boletin de Malariologia y Salud Ambiental	2
5	Effectiveness and Process Evaluation of Using Digital Health Technologies in Pharmaceutical Care in Low- and Middle-Income Countries: A Systematic Review of Quantitative and Qualitative Studies	Alfian et al	2023	Telemedicine journal and e-health : the official journal of the American Telemedicine Association	2
6	The effect of wellbeing mobile-applications on the mental and physical health of healthcare workers: A systematic review	Alford et al	2021	BJS Open	4
7	Blockchain Technology Effects on Healthcare Systems Using the IoT	Ajljabri et al	2023	Intelligent Internet of Things for Smart Healthcare Systems	2
8	A Systematic Review on Application of Data Mining Techniques in Healthcare Analytics and Data-Driven Decisions	Alloghani et al	2022	Studies in Computational Intelligence	3
9	An exploration of factors influencing health managers' acceptance of eHealth services in the Kingdom of Saudi Arabia	Alshahrani et al	2019	International Journal of Pharmacy Practice	4
10	Implementation strategies for telemental health: a systematic review	Appleton et al	2023	BMC health services research	4
11	Computer-generated reminders delivered on paper to healthcare professionals: Effects on professional practice and healthcare outcomes	Arditi et al	2017	The Cochrane Database of Systematic Reviews	2
12	The Efficacy of Digital Health Interventions for People with Traumatic Brain Injuries and their Carers: A Systematic Review	Avramovic et al	2022	Brain injury	4
13	A review of success/failure factors influencing healthcare personnel for telerehabilitation	Bahari et al	2019	6th International Conference on Research and Innovation in Information Systems: Empowering Digital Innovation, ICRIIS 2019	4
14	Barriers and facilitators to the availability of efficacious self-directed digital health tools for adults living with cancer and their caregivers: A systematic literature review and author survey study	Bamgboje-Ayodele et al	2021	Patient education and counseling	1
15	Benefits, drawbacks and challenges of social media use in Dermatology: a systematic review	Barrutia et al	2022	Journal of Dermatological treatment	2
16	Telework and Worker Health and Well-Being: A Review and Recommendations for Research and Practice	Beckel et al	2022	International journal of environmental research and public health	4
17	The use of social media in pharmacy practice and education	Benetoli et al	2015	Research in social & administrative pharmacy : RSAP	3
18	Safety and effectiveness of telementoring in surgery: a systematic review	Bilgic et al	2016	CMAJ. Canadian Medical Association Journal	4
19	Benchmark datasets driving artificial intelligence development fail to capture the needs of medical professionals	Blagec et al	2023	Journal of biomedical informatics	4
20	The effect of Electronic Health Records on the medical professional identity of physicians: A systematic literature review	Boonstra et al	2021	Procedia Computer Science	2
21	mHealth interventions to reduce maternal and child mortality in Sub-Saharan Africa and Southern Asia: A systematic literature review	Bossman et al	2022	Frontiers in global women's health	3
22	Creation of evidence-based resources to support pediatric healthcare professionals in adapting practices to include telehealth	Boychuck et al	2021	Developmental Medicine and Child Neurology	4
23	What unique knowledge and experiences do healthcare professionals have working in clinical informatics?	Brouat et al	2022	Informatics in medicine unlocked	4
24	Health care students experience of using digital technology in patient care: A scoping review of the literature	Brown Wilson et al	2020	Nurse education today	3
25	Artificial Intelligence for the Otolaryngologist: A State of the Art Review	Bur et al	2019	Otolaryngology - Head and Neck Surgery (United States)	4
26	The effectiveness of teleconsultations in primary care: systematic review	Carrillo de Albornoz et al	2022	Family practice	3
27	Telehealth as state response strategy: Systematic review	Celes et al	2018	Rev. Panam. Salud Publica Pan Am. J. Public Health	3
28	PNS42 Health Care Professionals and Mobile Applications: A Systematic Review	Chandran et al	2020	Value in Health Regional Issues	2
29	Computerised decision support systems for healthcare professionals: an interpretative review	Cresswel et al	2012	Inform in Prim Care	2

30	A systematic review of randomized controlled trials of telehealth and digital technology use by community pharmacists to improve public health	Crilly et al	2020	Pharmacy	3
31	Telemedicine versus face to face patient care: effects on professional practice and health care outcomes	Currell et al	2000	The Cochrane database of systematic reviews	3
32	Computer-aided anatomy recognition in intrathoracic and abdominal surgery: a systematic review	denBoer 2022	2022	Surgical Endoscopy	2
33	Using Machine Learning to Assess Physician Competence: A Systematic Review	Dias et al	2019	Academic medicine : journal of the Association of American Medical Colleges	3
34	Scoping review of pharmacist involvement using telehealth in transitions of care	Dixon et al	2017	Journal of the American Pharmacists Association	4
35	Telephone consultations for general practice: A systematic review	Downes et al	2017	Systematic Reviews	3
36	Potential of Internet of Medical Things (IoMT) applications in building a smart healthcare system: A systematic review	Dwivedi et al	2022	Journal of oral biology and craniofacial research	3
37	Who's using PDAs? Estimates of PDA use by health care providers: A systematic review of surveys	El Emam et al	2006	Journal of Medical Internet Research	4
38	The effectiveness of tele-triage during the COVID-19 pandemic: A systematic review and narrative synthesis	Farzandipour et al	2023	Journal of telemedicine and telecare	3
39	Hand hygiene teaching technologies for the healthcare team and caregivers: systematic review	Fernandes et al	2022	Bone marrow transplantation	2
40	Exploring digital health interventions to support community health workers in low-and-middle-income countries during the COVID-19 pandemic: A scoping review protocol	Feroz et al	2021	BMJ Open	4
41	Interventions to increase the use of electronic health information by healthcare practitioners to improve clinical practice and patient outcomes	Fiander et al	2015	The Cochrane database of systematic reviews	2
42	Using Mobile phone applications in engaging nurses for preventing helathcare-associated infections: A systematic review	Fithriyyah et al	2022	Nursing Practice today	2
43	Interactive telemedicine: effects on professional practice and health care outcomes	Flodgren et al	2015	The Cochrane database of systematic reviews	3
44	Efficacy of adaptive e-learning for health professionals and students: a systematic review and meta-analysis	Fontaine et al	2019	BMJ open	4
45	Nurse workarounds in the electronic health record: An integrative review	Fraczkowski et al	2020	J. Am. Med. Informatics Assoc.	3
46	The Effectiveness of Mobile-Health Technologies to Improve Health Care Service Delivery Processes: A Systematic Review and Meta-Analysis	Free et al	2013	PLoS Med.	2
48	Digital technologies in healthcare: What best practices and challenges?	Fusco et al	2021	Portuguese Journal of Public Health	4
49	Supporting health professionals through information and communication technologies: A systematic review of the effects of information and communication technologies on recruitment and retention	Gagnon et al	2011	Telemedicine. e-Health	2
50	Implementation frameworks for Artificial intelligence translation into Helathcare practive: Scoping review	Gama et al	2022	Journal of Medical Internet Research	2
51	Telemedicine solutions for clinical care delivery during COVID-19 pandemic: A scoping review	Ganjali et al	2022	Frontiers in public health	2
52	Acceptance of telemedicine technology among Physicians: A Systematic review	Garavand et al	2022	Informatics in medicine unlocked	2
53	Implentation studies for AI based tools in Helathcare should consider clinician competencies: Negative findings from a scoping review	Garvey 2022	2022	JMIR medical informatics	2
54	Considering Clinician competencies for the implementation of artificial intelligence - based tolls in healthcare: Findings from a scoping review	Garvey 2022	2022	JMIR medical informatics	2
55	Healthcare provider-targeted mobile applications to diagnose, screen, or monitor communicable diseases of public health importance in low- and middle-income countries: a systematic review	Geldsetzer et al	2022	medRxiv	4
56	Health information technology to facilitate communication involving health care providers, caregivers, and pediatric patients: a scoping review	Gentles et al	2010	Journal of medical Internet research	3
57	WhatsApp Messenger as an Adjunctive Tool for Telemedicine: An Overview	Giordano et al	2017	Interactive journal of medical research	2
58	Nurses' use of social media during the COVID 19 pandemic - A scoping review	Glasdam et al	2022	PloS one	2
59	Mobile technologies to support healthcare provider to healthcare provider communication and management of care	Gonçalves et al	2020	The Cochrane database of systematic reviews	2
60	Community health worker use of Smart devices for health promotion: Scoping review	Greuel et al	2023	JMIR mHealth and uHealth	2

61	TEleworRk-RelAted Stress (TERRA) as an emerging problem during the Covid-19 Pandemic: a Systematic Review	Gualano et al	2022	Safety and health at work	4
62	Application of Virtual Reality Technology in Clinical Practice, Teaching, and Research in Complementary and Alternative Medicine	Guan et al	2022	Evidence-based Complementary and Alternative Medicine	4
63	Parent and provider satisfaction of telehealt in pediatric surgical subspecialty care	Gudipudi et al	2022	Journal of telemedicine and telecare	2
64	Mobile-Social learning for continuing professional development in Low-and Middle-income countries: Integrative review	Guillaume et al	2022	JMIR medical education	2
65	Systematic review of evidence for the benefits of telemedicine	Hailey et al	2002	J Telemed Telecare	3
66	CORR Insights: How Satisfied Are Patients and Surgeons with Telemedicine in Orthopaedic Care During the COVID-19 Pandemic? A Systematic Review and Meta-analysis	Halai et al	2021	Clinical orthopaedics and related research	2
67	Social media use by health care professionals and trainees: A scoping review	Hamm et al	2013	Acad. Med.	3
68	The Application of Artificial Intelligence for Digital Imaging in the Operating Theatre: A Systematic Review and Narrative Synthesis	Hardacre et al	2022	British Journal of Surgery	3
69	Machine learning to guide clinical decision-making in abdominal surgery-a systematic literature review	Henn et al	2022	Langenbeck's archives of surgery	3
70	Clinician behaviors in telehealth care delivery: a systematic review	Henry et al	2017	Adv. Health Sci. Educ.	3
71	Examining the effectiveness of Web-based interventions to enhance resilience in Health care Professionals: Systematic review	Henshall et al	2022	JMIR medical education	2
72	Physician Satisfaction With Telehealth: A Systematic Review and Agenda for Future Research	Hoff et al	2022	Quality management in health care	4
73	Effects of computer-based clinical decision support systems on physician performance and patient outcomes: a systematic review	Hunt et al	1998	JAMA	4
74	Virtual and Augmented Reality in Neurosurgery: The Evolution of its Application and Study Designs	Jean, W. C.	2022	World neurosurgery	3
75	Alert fatigue and errors caused by technology: A scoping review and introduction to the flow of cognitive processing model	Joseph et al	2021	Knowledge Management and E-Learning	4
76	Promoting Physical Activity and Weight Loss With mHealth Interventions Among Workers: Systematic Review and Meta-analysis of Randomized Controlled Trials	Jung et al	2022	JMIR mHealth and uHealth	3
77	Virtual Reality Simulation for Disaster Preparedness Training in Hospitals: Integrated Review	Jung, Y.	2022	Journal of medical Internet research	3
78	Use of Telemedicine to Enhance Pharmacist Services in the Nursing Facility	Kane-Gill et al	2017	The Consultant pharmacist : the journal of the American Society of Consultant Pharmacists	3
79	Evaluation criteria ofr the effects of Decision support integrated into computerized provider order entry system: A scoping review	Karajuzadeh 2022	2022	Shiraz E Medical Journal	2
80	eHealth or e-Chaos: The use of Digital Health Interventions for Health Systems Strengthening in sub-Saharan Africa over the last 10 years: A scoping review	Karamagi et al	2022	Journal of global health	4
81	Physician leadership in e-health? A systematic literature review	Keijser et al	2016	Leadership in health services (Bradford, England)	4
82	Artificial Intelligence and Internet of Things (AI-IoT) Technologies in Response to COVID-19 Pandemic: A Systematic Review	Khan et al	2022	IEEE Access	4
83	Advanced Medication Reconciliation: A Systematic Review of the Impact on Medication Errors and Adverse Drug Events Associated with Transitions of Care	Killin et al	2021	Joint Commission Journal on Quality and Patient Safety	3
84	Examining mental workload relating to digital heal technologies in health care: Systematic review	Kremer et al	2022	Journal of Medical Internet Research	2
85	Leadership in the context of digital health services: A concept analysis	Laukka et al	2022	Journal of nursing management	3
86	3.129 The Effect of Mobile Digital Interventions on Mental Health Measures During the COVID-19 Pandemic: A Systematic Review of Controlled Studies	Lee et al	2022	Journal of the American Academy of Child and Adolescent Psychiatry	3
87	Tools to assess the trustworthiness of evidence-based point-of-care information for health care professionals: Systematic review	Lenaerts et al	2020	J. Med. Internet Res.	3
88	Exploration of implementation, financial and technical considerations within allied health professional (AHP) telehealth consultation guidance: a scoping review including UK AHP professional bodies‚Äô guidance	Leone et al	2021	BMJ Open	3
89	How mHealth Can Contribute to Improving the Continuum of Care: A Scoping Review Approach to the Case of Human Immunodeficiency Virus in Sub-Saharan Africa	Lepere et al	2022	Public health reviews	3
90	Electronic medical record-related burnout in healthcare providers: A scoping review of outcomes and interventions	Li et al	2022	BMJ Open	

91	Digital technologies for health workforce development in Low-and middle-income countries: A Scoping review	Long et al	2018	Global health, science and practice	2
92	Effects of e-Health interventions on stress reduction and mental health promotion in healthcare professionals: A systematic review	López-Del-Hoyo et al	2023	Journal of clinical nursing	2
93	Mobile Applications for Caregivers of Individuals with Chronic Conditions and/or Diseases: Quantitative Content Analysis	Lorca-Cabrera et al	2021	International journal of medical informatics	3
94	Health care professionals' experiences and perspectives on using telehealth for home-based palliative care: Protocol for a scoping review	Lundereng et al	2021	JMIR Res. Prot.	4
95	The effect of online health information seeking on physician-patient relationships: Systematic review	Luo et al	2022	Journal of Medical Internet Research	2
96	Influence of Health Social Networks on Healthcare: A Systematic Literature Review	Makena et al	2022	EPIC series in computing	4
97	Health information exchange policy and standards for digital health systems in africa: A systematic review	Mamuye et al	2022	PLOS digital health	4
98	Internet of Things Adoption for Saudi Healthcare Services	Masmali et al	2021	Pacific Asia Journal of the Association for Information Systems	3
99	Digital health technologies for osteopaths and allied healthcare service providers: A scoping review	Mastronardo et al	2021	Int. J. Ostheopath. Med.	3
100	Scoping review: Positive and negative impact of technology on clinicians	McBride et al	2023	Nursing Outlook	2
101	Effects of computerised clinical decision support systems (CDSS) on nursing and allied health professional performance and patient outcomes: A systematic review of experimental and observational studies	Mebrahtu et al	2021	BMJ Open	2
102	Lessons and Implementation Challenges of Community Health Information System in LMICs: A Scoping Review of Literature	Mekonnen et al	2022	Online journal of public health informatics	4
103	Teleconsultation in orthopaedic surgery: A systematic review and meta-analysis of patient and physician experiences	Melian et al	2022	Journal of telemedicine and telecare	3
104	Evidence of effectiveness of health care professionals using handheld computers: A scoping review of systematic reviews	Mickan et al	2013	J. Med. Internet Res.	2
105	Use of handheld computers in clinical practice: a systematic review	Mickan et al	2014	BMC medical informatics and decision making	2
106	A systematic review of the impact of health information technology on nurses' time	Moore et al	2020	Journal of the American Medical Informatics Association	2
107	Artificial intelligence and compassion in healthcare: A Systematic scoping review	Morrow et al	2023	Frontiers in Psychology	2
108	Experiences of patients and providers while using telemedicine in cancer care during COVID 19 pandemic: A systematic review and meta-synthesis of qualitative literature	Mostafaei et al	2022	Supportive care in cancer	2
109	Electronic health records and physician burnout: A scoping review	Muhiyaddin et al	2022	Studies in health technology and informatics	2
110	Computer-based clinical decision support for general practitioners	Murphy et al	2014	Family Practice	4
111	Impact of industry 4.0 on healthcare systems of low- and middle-income countries: a systematic review	Mwanza et al	2023	Health and technology	3
112	Health care professionals' perspectives on the secondary use of health records to improve quality and safety of care in England: Qualitative study	Neves et al	2019	J. Med. Internet Res.	4
113	Problematic problem diagnostics: Why digital health interventions for community health workers do not always achieve their desired impact	Newton-Lewis et al	2021	BMJ global health	4
114	The use of technology for urgent clinician to clinician communications: A systematic review of the literature	Nguyen et al	2015	Int. J. Med. Informatics	4
115	Health information communication technology evaluation frameworks for pharmacist prescribing: A systematic scoping review	Ogundipe et al	2023	Research in social & administrative pharmacy : RSAP	4
116	HOSPITAL MANAGERS' NEED FOR INFORMATION ON HEALTH TECHNOLOGY INVESTMENTS	Olholm et al	2015	International journal of technology assessment in health care	2
117	Effect of simulation on stress, anxiety and self confidene in nursing students: Systematic review with meta-analysis and meta-regression	Oliviera et al	2022	International journal of nursing studies 1	
118	Views of nurses and other health and social care workers on the use of assistive humanoid and animal-like robots in health and social care: a scoping review	Papadopoulos et al	2018	Contemporary nurse	3
119	Digital Health Interventions by Clinical Pharmacists: A Systematic Review	Park et al	2022	International Journal of Environmental Research and Public Health	2
120	Economic Evaluation of Pharmacist-Led Digital Health Interventions: A Systematic Review	Park et al	2022	International journal of environmental research and public health	3
121	HTA58 A Systematic Review of Digital Technology Use By Clinical Pharmacists	Park et al	2022	Value in Health	4

122	Potential of big data for healthcare and pharmaceutical professionals skills development	Pesqueira et al	2021	Top 10 Challenges of Big Data Analytics		3
123	Digital Mental Health Tools for Caregivers of Older Adults-A Scoping Review	Petrovic et al	2020	Frontiers in public health		4
124	How does telemonitoring impact medical education within the surgical field? A scoping review	Pfenning et al	2022	American journal of surgery	1	
125	The impact of mobile handheld technology on hospital physicians' work practices and patient care: a systematic review	Prgomet et al	2009	JAMIA		2
126	Applications of machine learning in routine laboratory medicine: Current state and future directions	Rabbani et al	2022	Clinical biochemistry		4
127	Cloud healthcare services: A comprehensive and systematic literature review	Rahimi et al	2022	Transactions on Emerging Telecommunications Technologies		3
128	Effects of computerized decision support systems on nursing performance and patient outcomes: a systematic review	Randell et al	2007	Journal of health services research & policy		3
129	A scoping review of applications of the Consolidated Framework for Implementation Research (CFIR) to telehealth service implementation initiatives	Rangachari et al	2022	BMC health services research		3
130	COVID-19 detection empowered with machine learning and deep learning techniques: A systematic review	Rehman et al	2021	Appl. Sci.		3
131	A systematic review and meta-analysis of online versus alternative methods for training licensed health care professionals to deliver clinical interventions	Richmond et al	2017	BMC Med. Educ.		3
132	Interventions to support the mental health and wellbeing of front line healthcare workers in hospitals during pandemics: an evidence review and synthesis	Robins-Browne et al	2022	BMJ Open	1	
133	Videoconference compared to telephone in healthcare delivery: A systematic review	Rush et al	2018	Int. J. Med. Informatics		2
134	Network approaches and interventions in healthcare settings: A systematic scoping review	Saatchi et al	2023	PloS one		3
135	Reviews of the implications of VR/AR health care applications in terms of organizational and societal change	Sharif et al	2018	Emerging Technologies for Health and Medicine: Virtual Reality, Augmented Reality, Artificial Intelligence, Internet of Things, Robotics, Industry 4.0		4
136	Implementation of Virtual Communities of Practice in Healthcare to Improve Capability and Capacity: A 10-Year Scoping Review	Shaw et al	2022	International journal of environmental research and public health		3
137	Impact of Internet-Based Interventions on Caregiver Mental Health: Systematic Review and Meta-Analysis	Sherifali et al	2018	Journal of medical Internet research		4
138	The effectiveness of internet-based e-learning on clinician behavior and patient outcomes: a systematic review protocol	Sinclair et al	2015	JBI Database System Rev Implement Rep		3
139	Determining if Telehealth Can Reduce Health System Costs: Scoping Review	Snoswell et al	2020	J. Med. Internet Res.		2
140	The Effectiveness of mHealth and eHealth Tools in Improving Provider Knowledge, Confidence, and Behaviors Related to Cancer Detection, Treatment, and Survivorship Care: a Systematic Review	Soloe et al	2021	J. Cancer Educ.		2
141	Effects of telehealth by allied health professionals and nurses in rural and remote areas: A systematic review and meta-Analysis	Speyer et al	2018	J. Rehabil. Med.		3
142	The introduction of computerized physician order entry and change management in a tertiary pediatric hospital	Staley et al	2005	Pediatrics		4
143	The review process used by US health care plans to evaluate new medical technology for coverage	Steiner et al	1996	Journal of general internal medicine		4
144	Development and Evaluation of Health Recommender Systems: Systematic Scoping Review and Evidence Mapping	Sun et al	2023	Journal of medical Internet research		3
145	An innovation involving self-surveillance and serious gaming to increase smoking quit rate: Protocol for a pilot randomized controlled trial	Tan et al	2021	Tobacco preventoin and cessation		4
146	Perceived Barriers and Facilitators of Using Synchronous Telerehabilitation of Physical and Occupational Therapy in Musculoskeletal Disorders: A Scoping Review	Tao et al	2022	medRxiv		4
147	E-learning in orthopedic surgery training: A systematic review	Tarpada et al	2016	Journal of Orthopaedics		4
148	Digital serious games in developing nursing clinical competence: A systematic review and meta-analysis	Thangavelu et al	2022	Nursing education today		2
149	Telemedicine and Pancreatic Cancer: A Systematic Review	Tripepi et al	2022	Telemedicine journal and e-health : the official journal of the American Telemedicine Association		4
150	Scoping the allied health eHealth landscape: A failed systematic review or a casualty of the business-government divide?	Voevodin, M.	2011	Australasian Medical Journal		2

151	Use of Telemedicine Technology among General Practitioners during COVID-19: A Modified Technology Acceptance Model Study in Poland	Walczak et al	2022	International journal of environmental research and public health	4
152	USE OF TELEMEDICINE IN GENERAL PRACTICE IN EUROPE SINCE THE COVID-19 PANDEMIC: A SCOPING REVIEW OF PATIENT AND PRACTITIONER PERSPECTIVES	Walley et al	2022	Irish journal of medical science	4
153	A Resilience Model for Moderating Outcomes Related to Electronic Medical Record Downtime	Walsh et al	2022	Studies in health technology and informatics	3
154	A systematic review of current teleophthalmology services in New Zealand compared to the four comparable countries of the United Kingdom, Australia, United States of America (UsA) and Canada	Walsh et al	2021	Clinical Ophthalmology	3
155	Using video consultation technology between care homes and health and social care professionals: a scoping review and interview study during COVID-19 pandemic	Warmoth et al	2022	Age and ageing	4
156	A Systematic Review of Research Studies Examining Telehealth Privacy and Security Practices used by Healthcare Providers	Watzlaf et al	2017	International journal of telerehabilitation	2
157	The impact of health information technology and e-health on the future demand for physician services	Weiner et al	2013	Health affairs (Project Hope)	4
158	Health Worker mHealth Utilization: A Systematic Review	White et al	2016	CIN Comput. Informatics Nurs.	2
159	A Scoping Review of Integrated Medical Devices and Clinical Decision Support in the Acute Care Setting	Withall et al	2022	Applied clinical informatics	1
160	The influence of web based tools on maternal and neonatal outcomes in pregnant adolescents or adolescent mothers: Mixed methods systematic review	Wu et al	2021	Journal of medical Internet research	2
161	The analyzation of change in documentation due to the introduction of Electronic patient records in hospitals - A systematic review	Wurster et al	2022	Journal of medical systems	2
162	Psychological Effects of Online-Based Mindfulness Programs during the COVID-19 Pandemic: A Systematic Review of Randomized Controlled Trials	Yeun et al	2022	International journal of environmental research and public health	3
163	A Systematic Review of Usefulness Design Goals of Occupational Mobile Health Apps for Healthcare Workers	Yingta et al	2021	Lect. Notes Comput. Sci.	4
164	Radiologists with assistance of deep learning can achieve overall accuracy of benign-malignant differentiation of musculoskeletal tumors comparable with that of pre-surgical biopsies in the literature	Zhao et al	2023	International journal of computer assisted radiology and surgery	3
165	The use of information and communication technology in healthcare to improve participation in everyday life: a scoping review	Zonneveld et al	2020	Disabil. Rehabil.	3

Legend: Reason of exclusion 1 – wrong intervention or platform was unclear; Reason 2 – the study did not provide any relevant barrier or facilitator that could be used in the project or did not provide any factor influencing healthcare providers; Reason 3 – targeted population was not healthcare providers; Reason 4 – study design used did not match with our inclusion criteria

Supplementary Information 2 – List of barriers and facilitators categorized in the review

Supplementary Information 2.1 - List of the 24 barriers categorized

Code Number	Outcome and finding
B1	Infrastructure and technical barriers
B2	Time and workload-related barriers
B3	Training and educational barriers
B4	Lack of supervisory support
B5	Ownership issues
B6	Health system-related barriers and financial barriers
B7	Cultural, social, and political barriers
B8	Personal and psychological barriers
B9	Simplicity of messages
B10	Interoperability issues and data incompatibility
B11	Communication issues
B12	Hierarchy-related barriers
B13	Organizational-related barriers
B14	Legal- and ethical- related barriers
B15	Interference between staff and patient interaction
B16	Lack of leadership and champions
B17	Fears on changing clinical roles and patterns of care, and existence of blurred professional boundaries and depiction of unprofessional behavior
B18	Disagreement with system-delivered decisions
B19	Hierarchy-related barriers
B20	Issues related with studies' design and quality of the evidence
B21	Excessive and unnecessary amount of data required
B22	Data security or equipment safety/security
B23	Lack of availability of high-quality data sets for training and validating algorithms and inadequate algorithms and computational power of artificial intelligence
B24	Shortage of interdisciplinary talents

Legend: In the code number column, B stands as Barrier (s)

Supplementary Information 2.2 - List of the 23 facilitators categorized

Code Number	Outcome and finding
F1	Involvement of healthcare professionals in process of development and implementation
F2	Intuitive navigation
F3	Offer training and educational activities
F4	Perceived usefulness and willingness
F5	Ease of use
F6	Existence of multiple functions of the technology
F7	Broader network coverage
F8	Financial support
F9	Adherence promotion
F10	Government and multisector incentives
F11	Reliability in the equipment
F12	Integration of the technology into the practice routine
F13	Leadership and local champion
F14	Maintenance of high-quality staff-patient interaction
F15	Pre-analysis of data and prior data processing
F16	Ownership and size of practice
F17	Previous computer experience
F18	Strong communication skills among healthcare professionals
F19	Transition planning
F20	Adequate number of personal which matches with local workload and workflow or assurance of quality of life to providers
F21	Technological innovativeness
F22	Conductance of more studies or confirmed study-based evidence of effectiveness of digital health technologies
F23	Focus in the patient and in the delivery of efficient delivery of care

Legend: In the code number column, F stands as Facilitator (s)

Supplementary Information 3. Figure legend and explanation of potential connections/relationships of identified barriers

Below, we coded each barrier to signalize and report potential connections between the extracted key terms, sentences, and identifiers. It is worthwhile mentioning that these thematic relationships are not limited in our analysis. Several links and connections can still be created and organized, based on a comprehensive, logic, and collaborative approach. Therefore, the presented connections were not fully exhausted.

Identifiers that have relationship with each other

a) 81B : 2B, 5B, 6B, and 7B
b) 82B and 83B : 9B
c) 58B : 3B
d) 27B : 30B
e) 19B: 28B, 58B
f) 30B : 25B, 33B, 58B, and 3B
g) 4B : 30B
h) 102B : 8B
i) 42B : 26B, 54B,
j) 46B : 34B, 42B
k) 39B : 50B
l) 38B : 72B, 73B
m) 45B : 67B
n) 78B : 98B : 106B
o) 15B : 60B, 61B, 65B
p) 77B : 41B,
q) 80B : 102B
r) 68B : 105B, 102B, 94B
s) 60B : 104B, 102B, 103B
t) 61B : 101B, 18B
u) 62B : 103B
v) 59B : 91B
w) 102B : 46B
x) 100B : 101B
y) 88B : 58B, 19B
z) 89B : 19B
aa) 86B : 32B
bb) 86B : 32B, 94B
cc) 101B : 29B
dd) 94B : 54B, 84B
ee) 49B : 93B
ff) 89B : 45B, 97BB
gg) 51B : 98B
hh) 84B : 37B, 99B,
ii) 16B : 101B
jj) 32B : 94B
kk) 104B : 82B
ll) 101B, 102B, 103B, 104B, and 105B : 108B
mm) 75B : 107B

Supplementary Information 4 – Protocol registered on PROSPERO

PROSPERO
International prospective register of systematic reviews

NHS
National Institute for Health Research

UNIVERSITY of York
Centre for Reviews and Dissemination

Systematic review

A list of fields that can be edited in an update can be found here

1. * Review title.

Give the title of the review in English

The impact of digital health solutions on health workers: an overview of systematic reviews

2. Original language title.

For reviews in languages other than English, give the title in the original language. This will be displayed with the English language title.

3. * Anticipated or actual start date.

Give the date the systematic review started or is expected to start.

21/01/2022

4. * Anticipated completion date.

Give the date by which the review is expected to be completed.

31/03/2022

5. * Stage of review at time of this submission.

This field uses answers to initial screening questions. It cannot be edited until after registration.

Tick the boxes to show which review tasks have been started and which have been completed.

Update this field each time any amendments are made to a published record.

The review has not yet started: Yes

Review stage	Started	Completed
Preliminary searches	No	No
Piloting of the study selection process	No	No
Formal screening of search results against eligibility criteria	No	No
Data extraction	No	No
Risk of bias (quality) assessment	No	No
Data analysis	No	No

Provide any other relevant information about the stage of the review here.

6. * Named contact.

The named contact is the guarantor for the accuracy of the information in the register record. This may be any member of the review team.

Israel Junior Borges do Nascimento

Email salutation (e.g. "Dr Smith" or "Joanne") for correspondence:

Dr Borges do Nascimento

7. * Named contact email.

Give the electronic email address of the named contact.

israeljrbn@gmail.com

8. Named contact address

Give the full institutional/organisational postal address for the named contact.

Medical Sciences Divisional Office - University of Oxford - Level 3, John Radcliffe Hospital Oxford OX3 9DU,

United Kingdom

9. Named contact phone number.

Give the telephone number for the named contact, including international dialling code.

+ 1 860 869 7285

10. * Organisational affiliation of the review.

Full title of the organisational affiliations for this review and website address if available. This field may be completed as 'None' if the review is not affiliated to any organisation.

Medical Sciences Divisional Office - University of Oxford

Organisation web address:

11. * Review team members and their organisational affiliations.

Give the personal details and the organisational affiliations of each member of the review team. Affiliation refers to groups or organisations to which review team members belong. **NOTE: email and country now MUST be entered for each person, unless you are amending a published record.**

Dr Israel Junior Borges do Nascimento. Medical Sciences Divisional Office - University of Oxford
Dr David Novillo Ortiz. Division of Country Health Policies and Systems, World Health Organization, regional office for Europe, Copenhagen, Denmark
Dr Hebatullah Mohamed Abdulazeem. Department of Sport and Health Science, Technische Universität München, Munich, Germany
Dr Lasse Østengaard. Cochrane Denmark Centre for Evidence-Based Medicine Odense (CEBMO) University Library of Southern Denmark
Dr Tomas Zapata Lopez. Division of Country Health Policies and Systems, World Health Organization,

regional office for Europe, Copenhagen, Denmark

12. * Funding sources/sponsors.

Details of the individuals, organizations, groups, companies or other legal entities who have funded or sponsored the review.

None

Grant number(s)

State the funder, grant or award number and the date of award

13. * Conflicts of interest.

List actual or perceived conflicts of interest (financial or academic).

None

14. Collaborators.

Give the name and affiliation of any individuals or organisations who are working on the review but who are not listed as review team members. **NOTE: email and country must be completed for each person, unless you are amending a published record.**

15. * Review question.

State the review question(s) clearly and precisely. It may be appropriate to break very broad questions down into a series of related more specific questions. Questions may be framed or refined using PI(E)COS or similar where relevant.

What is the impact of digital health solutions on health workers? What are the most solid evidence effects associated with of the use digital health technologies on the overall health worker? What are the main opportunities and challenges associated with the use of digital health solutions in health workers? Are there recommendations to be developed in the adoption and implementation of digital health solutions by health workers?

16. * Searches.

State the sources that will be searched (e.g. Medline). Give the search dates, and any restrictions (e.g. language or publication date). Do NOT enter the full search strategy (it may be provided as a link or attachment below.)

Based on a search strategy created by an experienced librarian, we will retrieve systematic reviews from five scientific databases: Cochrane Library (i.e., Cochrane Database of Systematic Reviews), Embase, Epistemonikos, MEDLINE, and Scopus. The planned retrieval date is December 23, 2021. We will not impose any language restriction on the obtained publications and for the purpose of this study, a systematic review will be defined as a review study, which followed evidence-based medicine related guidelines, and which considered at least 2 databases for its systematic search. Furthermore, the study should describe the search strategy used throughout the study idealization and should present a clear methodology for study selection and data extraction. We will include only systematic reviews that precisely analyze the current evidence associated with the use of digital health solutions in health workers, regardless the type of

outcomes assessed, and the type of primary studies included. We will exclude preprints, as well as unpublished data, and narrative or literature reviews. As part of a supplementary search, we will look at primarily selected papers references' lists and for the first 100 Google Scholar search results.

17. URL to search strategy.

Upload a file with your search strategy, or an example of a search strategy for a specific database, (including the keywords) in pdf or word format. In doing so you are consenting to the file being made publicly accessible. Or provide a URL or link to the strategy. Do NOT provide links to your search **results**.

Alternatively, upload your search strategy to CRD in pdf format. Please note that by doing so you are consenting to the file being made publicly accessible.

Do not make this file publicly available until the review is complete

18. * Condition or domain being studied.

Give a short description of the disease, condition or healthcare domain being studied in your systematic review.

Digital health solutions, Health workers, Systematic review, Evidence-based medicine.

19. * Participants/population.

Specify the participants or populations being studied in the review. The preferred format includes details of both inclusion and exclusion criteria.

Studies will be included in which health workers (either from the first, secondary, or tertiary level of care) are the focus of eligible systematic reviews.

20. * Intervention(s), exposure(s).

Give full and clear descriptions or definitions of the interventions or the exposures to be reviewed. The preferred format includes details of both inclusion and exclusion criteria.

We will include systematic reviews that directly evaluate the effect of digital health solutions on health workers.

21. * Comparator(s)/control.

Where relevant, give details of the alternatives against which the intervention/exposure will be compared (e.g. another intervention or a non-exposed control group). The preferred format includes details of both inclusion and exclusion criteria.

We will include systematic reviews that potentially compare digital health solutions/technologies in health workers to non-health workers. However, if a specific systematic review primarily did not focus on the existence of any comparator or controlling factor, we will consider this study for inclusion.

22. * Types of study to be included.

Give details of the study designs (e.g. RCT) that are eligible for inclusion in the review. The preferred format includes both inclusion and exclusion criteria. If there are no restrictions on the types of study, this should be stated.

Systematic reviews (with or without meta-analysis), that were published in peer-reviewed journal. Moreover,

we will consider the inclusion of scoping reviews, depending of the existence and report of adequately methodological characteristics (such as the existence of search strategies, a comprehensive study selection criteria, and a clear data summarization). We will not restrict this overview of systematic reviews based on the type of primary studies; therefore, if a certain systematic review includes randomized trials, observational studies, or mixed-method studies, it will be deemed eligible.

23. Context.

Give summary details of the setting or other relevant characteristics, which help define the inclusion or exclusion criteria.

Over the last number of years, several digital solutions have emerged as a significant and impactful alternative in human life. Precisely, these interventions have gained a wider space in the healthcare settings, particularly among health workers. To the best of our knowledge, no previous systematic review has been taken in place to understand the impact of digital health solutions in health workers, neither to assess the current evidence of its effects in the healthcare workplace. Therefore, in this systematic review we aim to collate, analyze, and verify the available evidence of the impact of these digital solutions in health workers, as well as to identify the main challenges, opportunities, and raise recommendations for the use of digital solutions by health workers.

2[illegible]. Main outcome(s).

Give the pre-specified main (most important) outcomes of the review, including details of how the outcome is defined and measured and when these measurement are made, if these are part of the review inclusion criteria.

[illegible] solutions or technologies on health workers (beneficial, negative, or any additional description of general effectiveness);

2. The awareness, feasibility, and acceptability of health workers of these digital interventions (using validated or non-validated structure/semi-structure questionnaire).

Moreover, we will analyze the potential effectiveness of digital solutions in improving clinical knowledge (i.e, improvement of delivered care, achievement of decreasing waiting time for medical procedures or medical evaluation, and so forth), staff behaviors, and practices on healthcare.

Furthermore, we will identify opportunities and challenges and develop recommendations in the adoption and implementation of digital health solutions by health workers. In addition, we plan to obtain studies that evaluate the social, economic, or medical impact of these solutions in the public health system in general (not only focused on health workers, but also in the functioning of the health environment).

Measures of effect

Please specify the effect measure(s) for you main outcome(s) e.g. relative risks, odds ratios, risk difference, and/or 'number needed to treat.

25. Additional outcome(s).

List the pre-specified additional outcomes of the review, with a similar level of detail to that required for main outcomes. Where there are no additional outcomes please state 'None' or 'Not applicable' as appropriate to the review

None.

Measures of effect

Please specify the effect measure(s) for you additional outcome(s) e.g. relative risks, odds ratios, risk difference, and/or 'number needed to treat.

26. * Data extraction (selection and coding).

Describe how studies will be selected for inclusion. State what data will be extracted or obtained. State how this will be done and recorded.

The results from the literature search performed by the information specialist will be uploaded to the online software, Covidence to allow inter-investigators collaboration. After duplicates removal, two independent reviewers will assess titles and abstracts to filter the studies that are most likely to be included. Afterwards, two reviewers will carry out full-text screening based on the beforementioned inclusion criteria. We will record the reasons for exclusion of each ineligible studies in this stage and report the justifications for exclusion in the final manuscript. We will resolve discrepancies either discussing between two reviewers or by the judgment of a third party. After categorization of finally included studies, two investigators will extract available data and investigate the methodological quality using an appropriate tool. If any discrepancy is noted at this stage, it will be resolved by consensus or a third reviewer. We will extract the following information from the selected studies: 1. general information (publication year, country, journal name, journal impact factor, disclosure of competing interests, and type or feature evaluated from the systematic review) 2. overall impact of digital solution in health worker (either qualitative or quantitative); 3. specific applicability of the digital solution in health worker environment; 4. the overall effect of digital health solutions in health worker (regardless the main focus from the eligible record); 5. main challenges, opportunities, and future recommendation from included reviews; and 6. limitations from included reviews. We will not restrict the data potentially available for extraction in these six main items. Therefore, if we identify any type of pattern among included reviews that might be relevant for inclusion, we will make the data displayed and will make a specific statement in the final publication regarding the addition of these outcome(s).

27. * Risk of bias (quality) assessment.

State which characteristics of the studies will be assessed and/or any formal risk of bias/quality assessment tools that will be used.

The methodological assessment of included systematic reviews will be based on the "Assessing the Methodological Quality of Systematic Reviews 2" tool. Overall, 16 critical domains are evaluated online, and a final rating is displayed by the software algorithm.

28. * Strategy for data synthesis.

Describe the methods you plan to use to synthesise data. This **must not be generic text** but should be

specific to your review and describe how the proposed approach will be applied to your data. If meta-analysis is planned, describe the models to be used, methods to explore statistical heterogeneity, and software package to be used.

We do not intend to perform a meta-analysis in the overview of systematic reviews. We will qualitatively describe the general characteristics of the included studies in summary tables using measures tendencies/units (primarily extracted by two reviews, using Microsoft Excel software). In addition, we plan to combine quantitative data into infographics, tables, or text-based medias to summarize the main results of each included systematic review. Therefore, a narrative synthesis is more likely to be created, in order to demonstrate the main findings of included studies, structured around the features targeted of primarily included systematic reviews. We will not restrict our synthetical analyses based on the number of final included studies. As the summary of effects among included studies can be varied and the measured outcomes might be multiples, we intend to include in our qualitative summary risk ratios, odds ratios, frequency values, and so forth.

29. * Analysis of subgroups or subsets.

State any planned investigation of 'subgroups'. Be clear and specific about which type of study or participant will be included in each group or covariate investigated. State the planned analytic approach.

None is intended to be performed as overviews of systematic reviews does not typically involves subgroups analyses.

30. * Type and method of review.

Select the type of review, review method and health area from the lists below.

Type of review

Cost effectiveness
No

Diagnostic
No

Epidemiologic
No

Individual patient data (IPD) meta-analysis
No

Intervention
No

Living systematic review
No

Meta-analysis
No

Methodology
Yes

Narrative synthesis
Yes

Network meta-analysis
No

Pre-clinical
No

Prevention
No

Prognostic
No

Prospective meta-analysis (PMA)
No

Review of reviews
Yes

Service delivery
No

Synthesis of qualitative studies
No

Systematic review
Yes

Other
No

Health area of the review

Alcohol/substance misuse/abuse
No

Blood and immune system
No

Cancer
No

Cardiovascular
No

Care of the elderly
No

Child health
No

Complementary therapies
No

COVID-19
No

Crime and justice
No

Dental
No

Digestive system

No

Ear, nose and throat
No

Education
No

Endocrine and metabolic disorders
No

Eye disorders
No

General interest
Yes

Genetics
No

Health inequalities/health equity
Yes

Infections and infestations
No

International development
No

Mental health and behavioural conditions
No

Musculoskeletal
No

Neurological
No

Nursing
No

Obstetrics and gynaecology
No

Oral health
No

Palliative care
No

Perioperative care
No

Physiotherapy
No

Pregnancy and childbirth
No

Public health (including social determinants of health)
Yes

Rehabilitation
No

Respiratory disorders
No

Service delivery
Yes

Skin disorders
No

Social care
No

Surgery
No

Tropical Medicine
No

Urological
No

Wounds, injuries and accidents
No

Violence and abuse
No

31. Language.

Select each language individually to add it to the list below, use the bin icon to remove any added in error.
English

There is not an English language summary

32. * Country.

Select the country in which the review is being carried out. For multi-national collaborations select all the countries involved.

Brazil
Denmark
England
Germany

33. Other registration details.

Name any other organisation where the systematic review title or protocol is registered (e.g. Campbell, or The Joanna Briggs Institute) together with any unique identification number assigned by them. If extracted data will be stored and made available through a repository such as the Systematic Review Data Repository (SRDR), details and a link should be included here. If none, leave blank.

34. Reference and/or URL for published protocol.

If the protocol for this review is published provide details (authors, title and journal details, preferably in Vancouver format)

Add web link to the published protocol.

Or, upload your published protocol here in pdf format. Note that the upload will be publicly accessible.

No I do not make this file publicly available until the review is complete

Please note that the information required in the PROSPERO registration form must be completed in full even if access to a protocol is given.

35. Dissemination plans.

Do you intend to publish the review on completion?

Yes

Give brief details of plans for communicating review findings.?

The paper will be potentially submitted to the Bulletin of the World Health Organization

(https://www.who.int/publications/journals/bulletin)

36. Keywords.

Give words or phrases that best describe the review. Separate keywords with a semicolon or new line. Keywords help PROSPERO users find your review (keywords do not appear in the public record but are included in searches). Be as specific and precise as possible. Avoid acronyms and abbreviations unless these are in wide use.

Digital health, health workers, systematic review, overview, evidence-based medicine.

37. Details of any existing review of the same topic by the same authors.

If you are registering an update of an existing review give details of the earlier versions and include a full bibliographic reference, if available.

38. * Current review status.

Update review status when the review is completed and when it is published.New registrations must be ongoing so this field is not editable for initial submission.
Please provide anticipated publication date

Review_Ongoing

39. Any additional information.

Provide any other information relevant to the registration of this review.

40. Details of final report/publication(s) or preprints if available.

Leave empty until publication details are available OR you have a link to a preprint (NOTE: this field is not editable for initial submission). List authors, title and journal details preferably in Vancouver format.

Give the link to the published review or preprint.

Supplementary Information 5 – Search strategies

Search details for
OVERVIEW OF SYSTEMATIC REVIEWS OF THE IMPACT OF DIGITAL HEALTH SOLUTIONS IN HEALTH WORKERS

By: Lasse Østengaard
The searches were conducted on the following dates:

- Initial search: January 21, 2022
- Updated search: March 1, 2023

Databases: Embase (Ovid), MEDLINE (Ovid), Cochrane library, Scopus and Epistemonikos.

Embase Classic+Embase 1947 to 2023 March 1

Embase Classic+Embase	
1	exp telehealth/
2	((Digital or mobile or smart) adj3 health).ti,ab.
3	(telehealth or telemedicine or telemonitoring).ti,ab.
4	exp mobile application/
5	internet/
6	exp mobile phone/
7	personal digital assistant/
8	Medical Informatics Applications/
9	computer assisted therapy/
10	(app or apps).ti,ab.
11	(online or internet or web* or digital* or tele* or google).ti.
12	((online or internet or web* or digital*) adj3 (based or application* or intervention* or program* or therap*)).ab.
13	(phone* or telephone* or smartphone* or cellphone* or smartwatch*).ti.
14	((phone* or telephone* or smartphone* or cellphone* or smartwatch*) adj3 (based or application* or intervention* or program* or therap*)).ab.
15	(mobile health or mhealth or m-health or ehealth or e-health or emental or e-mental).ti.
16	((mobile health or mhealth or m-health or ehealth or e-health or emental or e-mental) adj3 (based or application* or intervention* or program* or therap*)).ab.
17	(mobile* adj3 (based or application* or intervention* or device* or technolog*)).ti,ab.
18	(Connected Devices or Smart Devices or Digital Assistant).ti,ab.
19	exp medical informatics/ or exp electronic health record/ or electronic medical record/ or electronic patient record/
20	((Health or medical) adj3 (record* or informati* or data)).ti,ab.
21	(digital record* or personalized medicine or personalised medicine or interoperability).ti,ab.
22	exp artificial intelligence/ or exp machine learning/ or exp natural language processing/

23	((Artificial or machine or deep or hierarchical or ambient or comput*) adj3 (intelligence or learning)).ti,ab.
24	((Computer or automated) adj3 reasoning).ti,ab.
25	(Knowledge adj3 (acquisition or representation*)).ti,ab.
26	natural language processing.ti,ab.
27	(AI or NLP).ti,ab.
28	exp social media/
29	(social media or social network or Facebook or twitter or youtube or Instagram or flickr or Linkedin or blog* or on-line communit* or online communit* or wiki* or big data or open data or data mining or cloud or bluetooth or wearable* or wireless technology).ti,ab.
30	1 or 2 or 3 or 4 or 5 or 6 or 7 or 8 or 9 or 10 or 11 or 12 or 13 or 14 or 15 or 16 or 17 or 18 or 19 or 20 or 21 or 22 or 23 or 24 or 25 or 26 or 27 or 28 or 29
31	exp health care personnel/
32	((Health* or medical) adj3 (personnel or professional* or provider* or worker* or practitioner* or aide*)).ti,ab.
33	31 or 32
34	exp "systematic review"/ or exp meta analysis/
35	exp "systematic review (topic)"/ or exp "meta analysis (topic)"/
36	((systematic or scoping) adj3 (review* or overview*)).ti,ab.
37	(meta analy* or metaanaly* or meta-analy*).ti,ab.
38	34 or 35 or 36 or 37
39	30 and 33 and 38

Number of results from the first search in Embase: 6026
Number of results from the updated search in Embase: 1946
Total number of results from Embase 7972

Ovid MEDLINE(R) ALL 1946 to March 1, 2023

Ovid MEDLINE(R) ALL	
1	exp Telemedicine/
2	((Digital or mobile or smart) adj3 health).ti,ab.
3	(telehealth or telemedicine or telemonitoring).ti,ab.
4	Mobile Applications/
5	exp Internet/
6	exp Cell Phone/
7	exp Computers, Handheld/
8	Medical Informatics Applications/
9	Therapy, Computer-Assisted/
10	(app or apps).ti,ab.
11	(online or internet or web* or digital* or tele* or google).ti.

12	((online or internet or web* or digital*) adj3 (based or application* or intervention* or program* or therap*)).ab.
13	(phone* or telephone* or smartphone* or cellphone* or smartwatch*).ti.
14	((phone* or telephone* or smartphone* or cellphone* or smartwatch*) adj3 (based or application* or intervention* or program* or therap*)).ab.
15	(mobile health or mhealth or m-health or ehealth or e-health or emental or e-mental).ti.
16	((mobile health or mhealth or m-health or ehealth or e-health or emental or e-mental) adj3 (based or application* or intervention* or program* or therap*)).ab.
17	(mobile* adj3 (based or application* or intervention* or device* or technolog*)).ti,ab.
18	(Connected Devices or Smart Devices or Digital Assistant).ti,ab.
19	exp Medical Informatics/ or exp Medical Records Systems, Computerized/
20	((Health or medical) adj3 (record* or informati* or data)).ti,ab.
21	(digital record* or personalized medicine or personalised medicine or interoperability).ti,ab.
22	exp Artificial Intelligence/
23	((Artificial or machine or deep or hierarchical or ambient or comput*) adj3 (intelligence or learning)).ti,ab.
24	((Computer or automated) adj3 reasoning).ti,ab.
25	(Knowledge adj3 (acquisition or representation*)).ti,ab.
26	natural language processing.ti,ab.
27	(AI or NLP).ti,ab.
28	exp Social Media/
29	(social media or social network or Facebook or twitter or youtube or Instagram or flickr or Linkedin or blog* or on-line communit* or online communit* or wiki* or big data or open data or data mining or cloud or bluetooth or wearable* or wireless technology).ti,ab.
30	1 or 2 or 3 or 4 or 5 or 6 or 7 or 8 or 9 or 10 or 11 or 12 or 13 or 14 or 15 or 16 or 17 or 18 or 19 or 20 or 21 or 22 or 23 or 24 or 25 or 26 or 27 or 28 or 29
31	exp Health Personnel/
32	((Health* or medical) adj3 (personnel or professional* or provider* or worker* or practitioner* or aide*)).ti,ab.
33	31 or 32
34	exp "systematic review"/ or exp meta-analysis/
35	exp Systematic Reviews as Topic/ or exp Meta-Analysis as Topic/
36	((systematic or scoping) adj3 (review* or overview*)).ti,ab.
37	(meta analy* or metaanaly* or meta-analy*).ti,ab.
38	34 or 35 or 36 or 37
39	30 and 33 and 38

Number of results from the first search in MEDLINE: 3021
Number of results from the updated search in MEDLINE: 639
Total number of results from MEDLINE 3660

Cochrane library

ID	Search
#1	MeSH descriptor: [Telemedicine] explode all trees
#2	(((Digital or mobile or smart) near/2 health)):ti,ab,kw
#3	(telehealth or telemedicine or telemonitoring):ti,ab,kw
#4	MeSH descriptor: [Mobile Applications] this term only
#5	MeSH descriptor: [Internet] explode all trees
#6	MeSH descriptor: [Cell Phone] explode all trees
#7	MeSH descriptor: [Computers, Handheld] explode all trees
#8	MeSH descriptor: [Medical Informatics Applications] this term only
#9	MeSH descriptor: [Therapy, Computer-Assisted] this term only
#10	(app or apps):ti,ab,kw
#11	(online or internet or web* or digital* or tele* or google):ti
#12	(((online or internet or web* or digital*) near/2 (based or application* or intervention* or program* or therap*))):ab
#13	(phone* or telephone* or smartphone* or cellphone* or smartwatch*):ti
#14	(((phone* or telephone* or smartphone* or cellphone* or smartwatch*) near/2 (based or application* or intervention* or program* or therap*))):ab
#15	("mobile health" or mhealth or m-health or ehealth or e-health or emental or e-mental):ti
#16	((("mobile health" or mhealth or m-health or ehealth or e-health or emental or e-mental) near/2 (based or application* or intervention* or program* or therap*))):ab
#17	((mobile* near/2 (based or application* or intervention* or device* or technolog*))):ti,ab,kw
#18	("Connected Devices" or "Smart Devices" or "Digital Assistant"):ti,ab,kw
#19	MeSH descriptor: [Medical Informatics] explode all trees
#20	MeSH descriptor: [Medical Records Systems, Computerized] explode all trees
#21	(((Health or medical) near/2 (record* or informati* or data))):ti,ab,kw
#22	("digital record*" or "personalised medicine" or "personalized medicine" or interoperability):ti,ab,kw
#23	MeSH descriptor: [Artificial Intelligence] explode all trees
#24	(((Artificial or machine or deep or hierarchical or ambient or comput*) near/2 (intelligence or learning))):ti,ab,kw
#25	(((Computer or automated) near/2 reasoning)):ti,ab,kw
#26	((Knowledge near/2 (acquisition or representation*))):ti,ab,kw
#27	("natural language processing"):ti,ab,kw
#28	(AI or NLP):ti,ab,kw
#29	MeSH descriptor: [Social Media] explode all trees
#30	("social media" or "social network" or Facebook or twitter or youtube or Instagram or flickr or Linkedin or blog* or "on-line communit*" or "online communit*" or wiki* or "big data" or "open data" or "data mining" or cloud or bluetooth or wearable* or "wireless technology"):ti,ab,kw
#31	#1 or #2 or #3 or #4 or #5 or #6 or #7 or #8 or #9 or #10 or #11 or #12 or #13 or #14 or #15 or #16 or #17 or #18 or #19 or #20 or #21 or #22 or #23 or #24 or #25 or #26 or #27 or #28 or #29 or #30
#32	MeSH descriptor: [Health Personnel] explode all trees

#33	(((Health* or medical) near/2 (personnel or professional* or provider* or worker* or practitioner* or aide*))):ti,ab,kw
#34	#32 or #33
#35	#31 AND #34
#36	#31 AND #34 in Cochrane Reviews

Number of results from the first search in Cochrane library: 146
Number of results from the updated search in Cochrane library: 4
Total number of results from Cochrane library 150

Scopus

	Search Terms
26	((TITLE-ABS-KEY (telehealth OR telemedicine OR telemonitoring)) OR (TITLE-ABS-KEY ((digital OR mobile OR smart) W/2 health)) OR (TITLE-ABS-KEY ("mobile applications" OR internet OR "cell phone" OR "handheld computer*" OR "computer-assisted therapy")) OR (TITLE-ABS-KEY (app OR apps)) OR (TITLE (online OR internet OR web* OR digital* OR tele* OR google)) OR (ABS ((online OR internet OR web* OR digital*) W/2 (based OR application* OR intervention* OR program* OR therap*))) OR (TITLE (phone* OR telephone* OR smartphone* OR cellphone* OR smartwatch*)) OR (ABS ((phone* OR telephone* OR smartphone* OR cellphone* OR smartwatch*) W/2 (based OR application* OR intervention* OR program* OR therap*))) OR (TITLE ("mobile health" OR mhealth OR m-health OR ehealth OR e-health OR emental OR e-mental)) OR (ABS (("mobile health" OR mhealth OR m-health OR ehealth OR e-health OR emental OR e-mental) W/2 (based OR application* OR intervention* OR program* OR therap*))) OR (TITLE-ABS-KEY (mobile* W/2 (based OR application* OR intervention* OR device* OR technolog*))) OR (TITLE-ABS-KEY ("connected devices" OR "smart devices" OR "digital assistant")) OR (TITLE-ABS-KEY ((health OR medical) W/2 (record* OR informati* OR data))) OR (TITLE-ABS-KEY ("digital record*" OR "personalized medicine" OR "personalised medicine" OR interoperability)) OR (TITLE-ABS-KEY ((artificial OR machine OR deep OR hierarchical OR ambient OR comput*) W/2 (intelligence OR learning))) OR (TITLE-ABS-KEY ((computer OR automated) W/2 reasoning)) OR (TITLE-ABS-KEY (knowledge W/2 (acquisition OR representation*))) OR (TITLE-ABS-KEY ("natural language processing")) OR (TITLE-ABS-KEY (ai OR nlp)) OR (TITLE-ABS-KEY ("social media" OR "social network" OR facebook OR twitter OR youtube OR instagram OR flickr OR linkedin OR blog* OR "on-line communit*" OR "online communit*" OR wiki* OR "big data" OR "open data" OR "data mining" OR cloud OR bluetooth OR wearable* OR "wireless

	technology"))) AND (TITLE-ABS-KEY ((health* OR medical) W/2 (personnel OR professional* OR provider* OR worker* OR practitioner* OR aide*))) AND ((TITLE-ABS-KEY ((systematic OR scoping) W/2 (review* OR overview*))) OR (TITLE-ABS-KEY ("meta analy*" OR metaanaly* OR meta-analy*))) ...View More
25	(TITLE-ABS-KEY ((systematic OR scoping) W/2 (review* OR overview*))) OR (TITLE-ABS-KEY ("meta analy*" OR metaanaly* OR meta-analy*))
24	TITLE-ABS-KEY ("meta analy*" OR metaanaly* OR meta-analy*)
23	TITLE-ABS-KEY ((systematic OR scoping) W/2 (review* OR overview*))
22	TITLE-ABS-KEY ((health* OR medical) W/2 (personnel OR professional* OR provider* OR worker* OR practitioner* OR aide*))
21	(TITLE-ABS-KEY (telehealth OR telemedicine OR telemonitoring)) OR (TITLE-ABS-KEY ((digital OR mobile OR smart) W/2 health)) OR (TITLE-ABS-KEY ("mobile applications" OR internet OR "cell phone" OR "handheld computer*" OR "computer-assisted therapy")) OR (TITLE-ABS-KEY (app OR apps)) OR (TITLE (online OR internet OR web* OR digital* OR tele* OR google)) OR (ABS ((online OR internet OR web* OR digital*) W/2 (based OR application* OR intervention* OR program* OR therap*))) OR (TITLE (phone* OR telephone* OR smartphone* OR cellphone* OR smartwatch*)) OR (ABS ((phone* OR telephone* OR smartphone* OR cellphone* OR smartwatch*) W/2 (based OR application* OR intervention* OR program* OR therap*))) OR (TITLE ("mobile health" OR mhealth OR m-health OR ehealth OR e-health OR emental OR e-mental)) OR (ABS (("mobile health" OR mhealth OR m-health OR ehealth OR e-health OR emental OR e-mental) W/2 (based OR application* OR intervention* OR program* OR therap*))) OR (TITLE-ABS-KEY (mobile* W/2 (based OR application* OR intervention* OR device* OR technolog*))) OR (TITLE-ABS-KEY ("connected devices" OR "smart devices" OR "digital assistant")) OR (TITLE-ABS-KEY ((health OR medical) W/2 (record* OR informati* OR data))) OR (TITLE-ABS-KEY ("digital record*" OR "personalized medicine" OR "personalised medicine" OR interoperability)) OR (TITLE-ABS-KEY ((artificial OR machine OR deep OR hierarchical OR ambient OR comput*) W/2 (intelligence OR learning))) OR (TITLE-ABS-KEY ((computer OR automated) W/2 reasoning)) OR (TITLE-ABS-KEY (knowledge W/2 (acquisition OR representation*))) OR (TITLE-ABS-KEY ("natural language processing")) OR (TITLE-ABS-KEY (ai OR nlp)) OR (TITLE-ABS-KEY ("social media" OR "social network" OR facebook OR twitter OR youtube OR instagram OR flickr OR linkedin OR blog* OR "on-line communit*" OR "online communit*" OR wiki* OR "big data" OR "open data" OR "data mining" OR cloud OR bluetooth OR wearable* OR "wireless technology"))

20	TITLE-ABS-KEY ("social media" OR "social network" OR facebook OR twitter OR youtube OR instagram OR flickr OR linkedin OR blog* OR "on-line communit*" OR "online communit*" OR wiki* OR "big data" OR "open data" OR "data mining" OR cloud OR bluetooth OR wearable* OR "wireless technology")
19	TITLE-ABS-KEY (ai OR nlp)
18	TITLE-ABS-KEY ("natural language processing")
17	TITLE-ABS-KEY (knowledge W/2 (acquisition OR representation*))
16	TITLE-ABS-KEY ((computer OR automated) W/2 reasoning)
15	TITLE-ABS-KEY ((artificial OR machine OR deep OR hierarchical OR ambient OR comput*) W/2 (intelligence OR learning))
14	TITLE-ABS-KEY ("digital record*" OR "personalized medicine" OR "personalised medicine" OR interoperability)
13	TITLE-ABS-KEY ((health OR medical) W/2 (record* OR informati* OR data))
12	TITLE-ABS-KEY ("connected devices" OR "smart devices" OR "digital assistant")
11	TITLE-ABS-KEY (mobile* W/2 (based OR application* OR intervention* OR device* OR technolog*))
10	ABS (("mobile health" OR mhealth OR m-health OR ehealth OR e-health OR emental OR e-mental) W/2 (based OR application* OR intervention* OR program* OR therap*))
9	TITLE ("mobile health" OR mhealth OR m-health OR ehealth OR e-health OR emental OR e-mental)
8	ABS ((phone* OR telephone* OR smartphone* OR cellphone* OR smartwatch*) W/2 (based OR application* OR intervention* OR program* OR therap*))
7	TITLE (phone* OR telephone* OR smartphone* OR cellphone* OR smartwatch*)
6	ABS ((online OR internet OR web* OR digital*) W/2 (based OR application* OR intervention* OR program* OR therap*))
5	TITLE (online OR internet OR web* OR digital* OR tele* OR google)
4	TITLE-ABS-KEY (app OR apps)
3	TITLE-ABS-KEY ("mobile applications" OR internet OR "cell phone" OR "handheld computer*" OR "computer-assisted therapy")
2	TITLE-ABS-KEY ((digital OR mobile OR smart) W/2 health)
1	TITLE-ABS-KEY (telehealth OR telemedicine OR telemonitoring)

Number of results from the first search in Scopus: 3820
Number of results from the updated search in Scopus: 952
Total number of results from Scopus 4772

Epistemonikos

Note to the database:
The database does not support proximity operators, controlled vocabulary (e.g. MeSH terms) and there is a limitation to the length of the search. Adjustments has been made to the search. In the update, the search was split up in two parts due to the length of the URL generated when searching in Epistemonikos.

Number of results from the first search in Epistemonikos: 2622
Number of results from the updated search in Epistemonikos: 793
Total number of results from Epistemonikos 3415

(title:("digital" OR tele* OR "mhealth" OR "m-health" OR "ehealth" OR "e-health" OR "emental" OR "e-mental" OR "internet" OR "mobile phone" OR "computer assisted therapy" OR "connected devices" OR "smart devices" OR "online" OR web* OR "google" OR phone* OR smartphone* OR cellphone* OR smartwatch* OR "mobile health" OR "smart health" OR "mobile based" OR "mobile application" OR "mobile applications" OR "mobile intervention" OR "mobile interventions" OR "mobile device" OR "mobile devices" OR "mobile technology" OR "mobile technologies" OR "app" OR "apps" OR "social media" OR "social network" OR "Facebook" OR "twitter" OR "youtube" OR "Instagram" OR "flickr" OR "Linkedin" OR blog* OR "on-line community" OR "on-line communities" OR "online community" OR "online communities" OR wiki* OR "big data" OR "open data" OR "data mining" OR cloud OR bluetooth OR wearable* OR "wireless technology" OR "health record" OR "health records" OR "medical record" OR "medical records" OR "health information" OR "medical information" OR "health data" OR "medical data" OR "personalized medicine" OR "interoperability" OR "natural language processing" OR "AI" OR "NLP" OR "artificial intelligence" OR "machine intelligence" OR "deep intelligence" OR "hierarchical intelligence" OR "ambient intelligence" OR "computer intelligence" OR "artificial learning" OR "machine learning" OR "deep learning" OR "hierarchical learning" OR "ambient learning" OR "computer learning" OR "computer reasoning" OR "automated reasoning" OR "knowledge acquisition" OR "knowledge representation" OR "knowledge representations") OR abstract:("digital" OR tele* OR "mhealth" OR "m-health" OR "ehealth" OR "e-health" OR "emental" OR "e-mental" OR "internet" OR "mobile phone" OR "computer assisted therapy" OR "connected devices" OR "smart devices" OR "online" OR web* OR "google" OR phone* OR smartphone* OR cellphone* OR smartwatch* OR "mobile health" OR "smart health" OR "mobile based" OR "mobile application" OR "mobile applications" OR "mobile intervention" OR "mobile interventions" OR "mobile device" OR "mobile devices" OR "mobile technology" OR "mobile technologies" OR "app" OR "apps" OR "social media" OR "social network" OR "Facebook" OR "twitter" OR "youtube" OR "Instagram" OR "flickr" OR "Linkedin" OR blog* OR "on-line community" OR "on-line communities" OR "online community" OR "online communities" OR wiki* OR "big

data" OR "open data" OR "data mining" OR cloud OR bluetooth OR wearable* OR "wireless technology" OR "health record" OR "health records" OR "medical record" OR "medical records" OR "health information" OR "medical information" OR "health data" OR "medical data" OR "personalized medicine" OR "interoperability" OR "natural language processing" OR "AI" OR "NLP" OR "artificial intelligence" OR "machine intelligence" OR "deep intelligence" OR "hierarchical intelligence" OR "ambient intelligence" OR "computer intelligence" OR "artificial learning" OR "machine learning" OR "deep learning" OR "hierarchical learning" OR "ambient learning" OR "computer learning" OR "computer reasoning" OR "automated reasoning" OR "knowledge acquisition" OR "knowledge representation" OR "knowledge representations")) AND (title:("health personnel" OR "healthcare personnel" OR "health professional" OR "health professionals" OR "healthcare professional" OR "healthcare professionals" OR "health provider" OR "health providers" OR "healthcare provider" OR "healthcare providers" OR "health worker" OR "health workers" OR "healthcare worker" OR "healthcare workers" OR "health practitioner" OR "health practitioners" OR "healthcare practitioner" OR "healthcare practitioners" OR "health aide" OR "health aides" OR "healthcare aide" OR "healthcare aides" OR "medical personnel" OR "medical professional" OR "medical professionals" OR "medical provider" OR "medical providers" OR "medical worker" OR "medical workers" OR "medical practitioner" OR "medical practitioners" OR "medical aide" OR "medical aides") OR abstract:("health personnel" OR "healthcare personnel" OR "health professional" OR "health professionals" OR "healthcare professional" OR "healthcare professionals" OR "health provider" OR "health providers" OR "healthcare provider" OR "healthcare providers" OR "health worker" OR "health workers" OR "healthcare worker" OR "healthcare workers" OR "health practitioner" OR "health practitioners" OR "healthcare practitioner" OR "healthcare practitioners" OR "health aide" OR "health aides" OR "healthcare aide" OR "healthcare aides" OR "medical personnel" OR "medical professional" OR "medical professionals" OR "medical provider" OR "medical providers" OR "medical worker" OR "medical workers" OR "medical practitioner" OR "medical practitioners" OR "medical aide" OR "medical aides")) AND (title:(((systematic OR scoping) AND (review* OR overview*)) OR ((meta analy*) OR metaanaly* OR meta-analy*)) OR abstract:(((systematic OR scoping) AND (review* OR overview*)) OR ((meta analy*) OR metaanaly* OR meta-analy*)))
